# Tailoring poplar lignin without yield penalty by combining a null and haploinsufficient *CINNAMOYL-CoA REDUCTASE2* allele

**DOI:** 10.1038/s41467-020-18822-w

**Published:** 2020-10-06

**Authors:** Barbara De Meester, Barbara Madariaga Calderón, Lisanne de Vries, Jacob Pollier, Geert Goeminne, Jan Van Doorsselaere, Mingjie Chen, John Ralph, Ruben Vanholme, Wout Boerjan

**Affiliations:** 1 Department of Plant Biotechnology and Bioinformatics, Ghent University, Technologiepark 71, 9052 Ghent, Belgium; 2 VIB Center for Plant Systems Biology, Technologiepark 71, 9052 Ghent, Belgium; 3 VIB Metabolomics Core, Technologiepark 71, 9052 Ghent, Belgium; 4 Higher Institute for Nursing and Biotechnology, VIVES University College, Wilgenstraat 32, 8800 Roeselare, Belgium; 5 Department of Biochemistry, University of Wisconsin-Madison, Madison, WI 53706 USA; 6 US Department of Energy, Great Lakes Bioenergy Research Center, Wisconsin Energy Institute, Madison, WI 53726 USA

**Keywords:** Molecular engineering in plants, CRISPR-Cas9 genome editing, Molecular engineering in plants, Cell wall

## Abstract

Lignin causes lignocellulosic biomass recalcitrance to enzymatic hydrolysis. Engineered low-lignin plants have reduced recalcitrance but often exhibit yield penalties, offsetting their gains in fermentable sugar yield. Here, CRISPR/Cas9-generated *CCR2*(−/*) line 12 poplars have one knockout *CCR2* allele while the other contains a 3-bp deletion, resulting in a 114I115A-to-114T conversion in the corresponding protein. Despite having 10% less lignin, *CCR2*(−/*) line 12 grows normally. On a plant basis, the saccharification efficiency of *CCR2*(−/*) line 12 is increased by 25–41%, depending on the pretreatment. Analysis of monoallelic *CCR2* knockout lines shows that the reduced lignin amount in *CCR2*(−/*) line 12 is due to the combination of a null and the specific haploinsufficient *CCR2* allele. Analysis of another *CCR2*(−/*) line shows that depending on the specific CCR2 amino-acid change, lignin amount and growth can be affected to different extents. Our findings open up new possibilities for stably fine-tuning residual gene function *in planta*.

## Abstract (web summary)

Plants with reduced amounts of lignin typically suffer from dwarfed growth, which offsets their gain in fermentable sugar yield. Here, the authors show that genome-edited poplar lines with a null and a haploinsufficient allele of *CINNAMOYL-COA REDUCTASE2* (*CCR2*) can be obtained that have a reduced lignin level and normal growth.

## Introduction

The lignin polymer provides strength and hydrophobicity to the plant cell wall and is generally derived from the monolignols coniferyl and sinapyl alcohol and low levels of *p*-coumaryl alcohol. Depending on the plant species, other monomers or derivatives may also contribute to the lignin polymer^1^. After polymerization in the cell wall, the monolignols produce guaiacyl (G), syringyl (S), and *p*-hydroxyphenyl (H) units, respectively^1^.

Engineering plants to deposit less lignin is a promising strategy to enable improved biomass processability. However, hurdles need to be overcome for the development of low-lignin elite clones for forestry applications. One hurdle is to reduce lignin amount in a stable way. For example, RNA interference (RNAi) was frequently used to downregulate the expression of lignin biosynthesis genes in poplar^2–5^. However, this method often results in unstable downregulation of the targeted genes^3–5^. As an illustration, the red xylem phenotype caused by reductions in CINNAMOYL-CoA REDUCTASE (CCR) activity, appeared in patches on debarked *CCR2*-downregulated poplar stems, as a consequence of the unequal levels of gene silencing in red versus white regions^3^. Unequal gene silencing levels even appeared between individual clones of the same *CCR2*-downregulated line^3^. A second hurdle is to reduce lignin amount without affecting plant development and biomass yield. For example, the *CCR2*-downregulated poplars with the highest levels of *CCR2* downregulation had up to 24% less lignin and an up to 104% increased enzymatic cellulose-to-glucose conversion without pretreatment^3^. Unfortunately, similar to many other plants yielding higher cellulose-to-glucose conversion levels^2,6–8^, these *CCR2*-downregulated poplars suffered from a reduction of up to 51% in biomass, (entirely) offsetting their gains in fermentable sugar yield^3^. Hence, for applications, a method is desired to make plants with a stable and fine-tuned lignin amount to still achieve higher sugar yields in all replicates, but without affecting growth.

To evaluate the specific role of *CCR2* in poplar, *CCR2*(−/−) null mutants are generated using CRISPR/Cas9. In addition to severely dwarfed *CCR2*(−/−) plants, a biallelically modified line has normal growth. Here, we show that this line, named *CCR2*(−/*) line 12, contains a knockout and a specific haploinsufficient *CCR2* allele (114I115A-to-114T amino-acid change in the corresponding CCR2 protein sequence) that results in a uniformly distributed red xylem phenotype, a 10% reduction in lignin amount and a 25 to 41% increase in saccharification efficiency on a plant basis, depending on the applied pretreatment. Analysis of another *CCR2*(−/*) line shows that, whereas multiple amino-acid changes in CCR2 can result in lower lignin content (to different extents), they will not all allow normal growth. We propose that *in planta* screening for combinations of a knockout and a haploinsufficient allele is a promising strategy to fine-tune the desired level of residual gene function.

## Results

### *CCR2*(−/*) line 12 grows normally while having red xylem

**Fig. 1:**
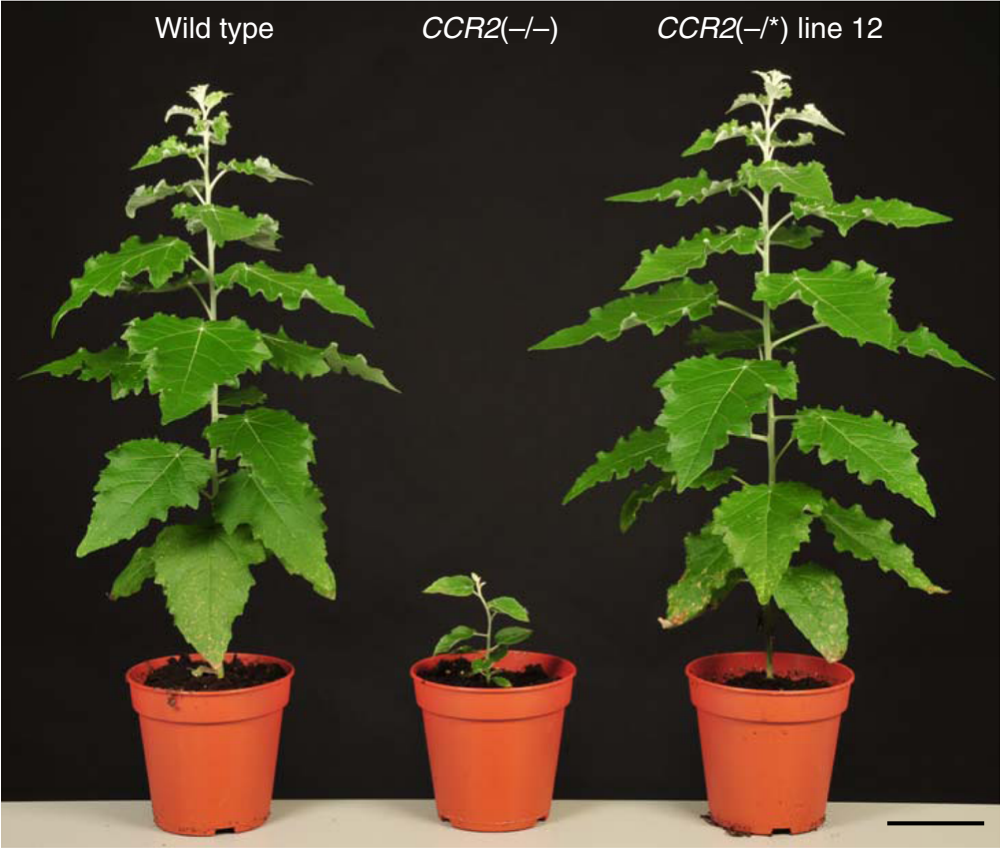
Phenotype of poplar containing biallelic *CCR2* mutations. Plants were grown for 11 weeks in the greenhouse. *CCR2*(−/−) and *CCR2*(−/*) line 12 poplars (*P. tremula* × *P. alba)* were generated via CRISPR/Cas9 using gRNA1 (targeting the third exon of both *CCR2* alleles). The status of the *CCR2* alleles present in *P. tremula* × *P. alba* is denoted between the parentheses; the first one represents that of the *P. tremula* allele, the second one that of the *P. alba* allele; −, knockout; *, protein-modified. The plants shown are representative of twenty biologically independent samples for wild type and *CCR2*(−/−). One plant was available for *CCR2*(−/*) line 12. Scale bar = 10 cm.

To evaluate the effect of fully knocking out *CCR2* on the phenotype of poplar, we generated 21 biallelically edited *CCR2* mutants in *Populus tremula* × *P. alba* by CRISPR/Cas9 using a gRNA (gRNA1) targeting the third exon of the *CCR2* gene (Supplementary Fig. 1a). The twenty lines that contained biallelic frameshift mutations in *CCR2*, *CCR2*(−/−) lines, were all severely dwarfed (Fig. 1; Supplementary Fig. 1b). Interestingly, one biallelic mutant line did not display observable growth perturbations (Fig. 1). *CCR2*(−/*) line 12 had a frameshift mutation (1-bp insertion) in the *P. tremula CCR2* allele, and a deletion of 3 bp in the *P. alba* *CCR2* allele, which resulted into a substitution of Ile114 and Ala115 for a Thr114 in the corresponding *P. alba* CCR2 protein sequence (Supplementary Figs. 1b and  2). The amino-acid change occurred in α4 of the CCR2 protein, but not in the active site, NAPD-binding domain, or substrate-binding pocket residues^9–11^.

**Fig. 2:**
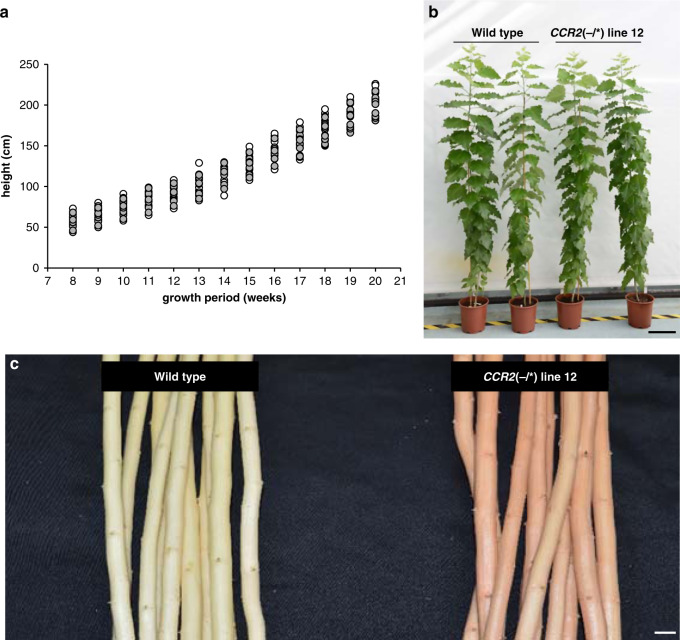
Phenotype of *CCR2*(−/*) line 12 poplar. Plants were grown for 20 weeks in the greenhouse. **a** Growth curve of wild type and *CCR2*(−/*) line 12. No significant differences in height were found between the wild type (individual values shown in white dots) and *CCR2*(−/*) line 12 (individual values shown in gray dots) at the 0.01 significance level (two-tailed Student’s *t*-test). For mean values and exact *P* values, see Supplementary Table 1. **b** Phenotype of representative wild type and *CCR2*(−/*) line 12 after growing for 20 weeks in the greenhouse. Scale bar = 20 cm. **c** Phenotype of debarked wild-type and *CCR2*(−/*) line 12 stems grown in the greenhouse for 20 weeks. *CCR2*(−/*) line 12 stems display the red xylem phenotype. Scale bar = 10 mm. Wild type, *n* = 10 biologically independent samples; *CCR2*(−/*) line 12, *n* = 11 biologically independent samples. The source data underlying (**a**) are provided as a Source data file.

To evaluate the growth and xylem phenotypes of *CCR2*(−/*) line 12, wild type, and *CCR2*(−/*) line 12 were clonally propagated to generate multiple biological replicates. The replicates were grown in the greenhouse for a period of 20 weeks. Plant height was followed weekly, and by the end of the growth period, the trees were harvested, and biomass parameters were determined. The stem height of *CCR2*(−/*) line 12 was equal to that of the wild type during the entire growth period (Fig. 2a, Supplementary Table 1). At harvest time, the *CCR2*(−/*) line 12 plants were morphologically indistinguishable from the wild type (Fig. 2b), and no differences in either stem diameter or fresh and dry stem weight were observed (Table 1).

**Table 1:** Biomass and cell wall composition of *CCR2*(−/*) line 12 stems.

	Wild type	*CCR2*(−/*) line 12	*P* value
Fresh weight with bark (g)	87.6 ± 23.9	87.0 ± 19.4	0.956
Fresh weight debarked (g)	57.4 ± 15.0	57.0 ± 13.1	0.940
Dry weight debarked (g)	18.8 ± 5.6	17.6 ± 4.6	0.582
Height (cm)	207.4 ± 14.2	198.2 ± 11.9	0.123
Diameter (mm)	11.4 ± 1.0	11.0 ± 0.8	0.427
CWR (% dry weight)	87.8 ± 0.7	87.4 ± 0.7	0.156
Cellulose (% CWR)	39.6 ± 3.8	39.5 ± 3.9	0.976
Matrix polysaccharides (%CWR)	37.8 ± 1.8	41.9 ± 3.1**	0.002
Total Klason lignin (% CWR)	31.1 ± 1.5	27.8 ± 0.9**	5.8 × 10^−6^
Acid-insoluble Klason lignin (%CWR)	29.4 ± 1.5	26.0 ± 0.9**	3.4 × 10^−6^
Acid-soluble Klason lignin (%CWR)	1.69 ± 0.10	1.82 ± 0.10**	0.008
Acetyl bromide lignin (%CWR)	17.1 ± 1.3	15.4 ± 0.7**	0.004
Thioacidolysis-released monomers			
H + G + S	15.9 ± 0.9	15.4 ± 0.5	0.092
%H	1.6 ± 0.2	0.3 ± 0.1**	5.0 × 10^−9^
%S	56.1 ± 0.5	56.7 ± 0.8	0.087
%G	42.3 ± 0.5	43.0 ± 0.8*	0.020
S/G	1.33 ± 0.03	1.32 ± 0.04	0.521
%β-*O*-4-FA-I	0.013 ± 0.003	0.073 ± 0.016**	1.6 × 10^−7^
%β-*O*-4-FA-II	0.006 ± 0.001	0.040 ± 0.005**	1.0 × 10^−9^
%*bis*-β-*O*-4-FA	0.107 ± 0.040	0.818 ± 0.223**	6.2 × 10^−7^
%G-aldehydes	1.0 ± 0.2	1.2 ± 0.1	0.097
NMR-derived aromatic units			
%H	0.54 ± 0.22	0.27 ± 0.06	0.113
%S	59.7 ± 0.7	56.2 ± 1.4*	0.021
%G	39.8 ± 0.6	43.5 ± 1.5*	0.016
S/G	1.50 ± 0.04	1.29 ± 0.08*	0.015
%*p*BA	6.3 ± 0.4	11.0 ± 0.6**	3.3 × 10^−4^
%FM	0.0	0.2***	
NMR-derived interunit linkages			
%β-Aryl ether (A)	83.7 ± 0.4	86.5 ± 1.3*	0.026
%Phenylcoumaran (B)	6.0 ± 0.3	5.9 ± 1.1	0.826
%Resinol (C)	10.2 ± 0.4	7.6 ± 0.2**	6.2 × 10^−4^

Plants were grown for 20 weeks in the greenhouse. Stem diameter was determined 3 cm above soil level. At the time of harvest, the height, fresh weight (without leaves; with or without bark), and diameter of the stem were measured. After drying the debarked stems for 2 weeks, the dry weight was determined. Cell wall residue (CWR) was determined after sequential extraction of dry debarked stem material. Crystalline cellulose content was determined by the Updegraff method and the mass loss during TFA extraction was used as an estimate of the amount of matrix polysaccharides. Lignin content was determined by the Klason and acetyl bromide methods. Lignin composition was determined by 2D HSQC NMR and by thioacidolysis. The sum of H, G, and S is expressed in μmol per g Klason lignin. %H + %G + %S = 100; other units are expressed versus H + G + S. *p*BA: *p*-hydroxybenzoate; FM: ferulic acid marker (see Supplementary Fig. 3). Lignin interunit types (A–C), from uncorrected volume-integrals only, are expressed on an A + B + C = 100% basis. The data represent means ± standard deviation. **P < 0.01, *P < 0.05, two-tailed Student’s *t*-test. The exact *P* value is shown in the table. For NMR, wild type and *CCR2*(−/*) line 12, *n* = 3 biologically independent samples. For all other analysis, wild type, *n* = 10 biologically independent samples and *CCR2*(−/*) line 12, *n* = 11 biologically independent samples. Source data are provided as a Source data file.

After debarking the harvested stems, the coloration of the xylem could be judged; whereas wild-type xylem had a white-to-beige color, *CCR2*(−/*) line 12 xylem displayed a uniform pink-to-red coloration (Fig. 2c). This xylem coloration is often observed in lignin-modified plants^3,4,12,13^ and suggested a modified lignin in *CCR2*(−/*) line 12 as compared to the wild type.

**Fig. 3:**
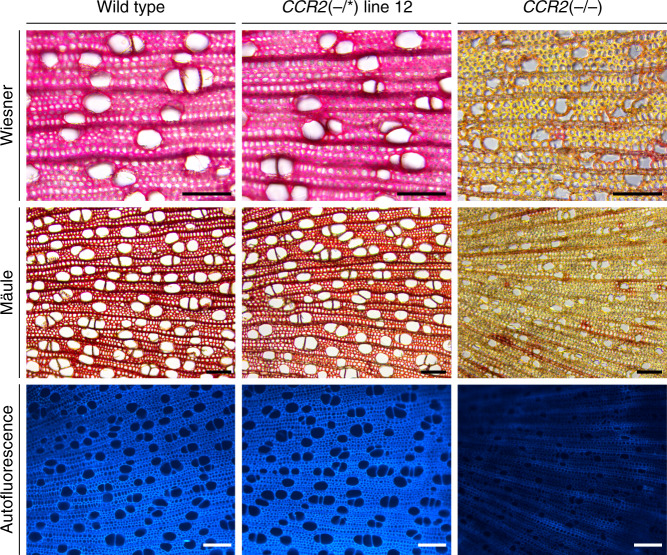
Xylem morphology in stems of *CCR2*(−/*) line 12 and *CCR2*(−/−) poplars. Plants were grown for 20 weeks in the greenhouse. Lignin was visualized using Mäule and Wiesner staining and via lignin autofluorescence (*λ*_ex_ = 365 nm, *λ*_em_ = 420–470 nm). Pictures were taken of the xylem tissue, with the pith localized to the left and the epidermis to the right. Images show representative pictures from five sections per sample and 3 biologically independent samples per line. Scale bar = 100 μm.

To examine the morphology of the xylem cells in the stem, cross-sections of wild type, *CCR2*(−/*) line 12, and *CCR2*(−/−) were observed via light microscopy after Wiesner and Mäule staining, and via fluorescence microscopy (Fig. 3). Wild-type and *CCR2*(−/*) line 12 stem sections were morphologically indistinguishable. They both had fiber and vessel cells with lignified cell walls, and the vessel cells were open. By contrast, *CCR2*(−/−) stems showed an overall reduction in lignin deposition in the cell walls of both fiber and vessel cells, and the vessel cells had an irregular shape.

### Altered lignin in *CCR2*(−/*) line 12

To evaluate the lignocellulosic biomass composition of *CCR2*(−/*) line 12 stems, the cell wall residue (CWR), the cellulose content, and the lignin content and composition of dried debarked stem material were determined (Table 1). CWR was prepared by applying a sequential extraction to remove soluble compounds from the stems. The fraction of CWR (as % of the dry weight) of *CCR2*(−/*) line 12 did not differ from that of the wild type. The cellulose content of the prepared CWRs was analyzed via the spectrophotometric Updegraff assay which showed that the crystalline cellulose content of *CCR2*(−/*) line 12 did not differ significantly from that of the wild type. The combined amount of matrix polysaccharides and amorphous cellulose—determined as the mass loss upon trifluoroacetic acid treatment—in the CWRs of *CCR2*(−/*) line 12 was increased by about 10% when compared to that of the wild type. Next, the fraction of lignin in the prepared CWRs was determined via the Klason and the acetyl bromide methods. Both methods showed that the total lignin amount of *CCR2*(−/*) line 12 was decreased by about 10% when compared to that of the wild type.

Lignin composition in the CWRs was evaluated via thioacidolysis, an analytical method that quantifies lignin units solely linked by β–*O–*4 bonds (Table 1). *CCR2*(−/*) line 12 lignins showed a trend towards releasing more monomers (H + G + S) when compared to wild-type lignins (*P* = 0.092), indicative for a slightly higher frequency of β–*O–*4 interunit bonds. In the wild type, H monomers constituted 1.6% of the total identified thioacidolysis-released units. In *CCR2*(−/*) line 12, the fraction of thioacidolysis-released H units was only 0.3%. The S/G ratio based on thioacidolysis-released monomers was equal for the wild type and *CCR2*(−/*) line 12. Incorporation of ferulic acid (FA), which is a known minor constituent of lignin, results in the release of three different units after thioacidolysis: β–*O–*4-FA-I and β–*O–*4-FA-II are derived from ferulic acid (or ferulate ester) starting units coupled via their *O–*4 position in β–*O–*4 interunit bonds, whereas *bis*-β–*O–*4-FA is derived from ferulic acid that has undergone a β–*O–*4 coupling twice at its β-position^14^. In agreement with previously reported results for plants deficient in CCR^3,5,14–16^, the relative abundance of all three thioacidolysis-released ferulic acid units was increased in *CCR2*(−/*) line 12 when compared to the wild type.

The lignin composition was also analyzed via two-dimensional ^1^H–^13^C heteronuclear single-quantum coherence (2D HSQC) nuclear magnetic resonance (NMR) on ball-milled whole-cell-wall material prepared from the stem (Table 1, Supplementary Fig. 3). By analyzing the aromatic regions of the 2D HSQC spectra, it is possible to profile differences in lignin monomeric composition irrespective of the interunit linkage distribution. Using NMR, the estimated relative frequency of H units showed no significant differences between wild type and *CCR2*(−/*) line 12. However, the determination of H units via NMR is neither sensitive nor accurate because the peaks used for their estimation are contaminated by those from phenylalanine^17^. In contrast to the results obtained by thioacidolysis, the S/G ratio was decreased by 14% in *CCR2*(−/*) line 12 when compared to the wild type. Similar to the results obtained from thioacidolysis, the ferulic acid marker peak was clearly detected in spectra from *CCR2*(−/*) line 12 wood, while being absent in spectra from the wild type. In addition, the NMR data enabled the relative measurement of *p*-hydroxybenzoates that acylate the sidechain γ-OH of G and, predominantly, S units in poplar. The relative frequency of these moieties was increased by 75% in *CCR2*(−/*) line 12 when compared to the wild type. This observation is in line with the fact that the biosynthesis of *p*-hydroxybenzoates, in contrast to that of H, G, and S units, is independent of CCR2 activity. The interunit linkage-type distributions were also deduced from the NMR spectra. The lignin of *CCR2*(−/*) line 12 showed an increase in the relative proportion of β-aryl ether (β–*O–*4) units, at the expense of resinol (β–β) units. The fraction of phenylcoumarans (β–5) did not differ between *CCR2*(−/*) line 12 and the wild type.

Finally, analysis by gel-permeation chromatography (GPC) showed that the molecular weight of *CCR2*(−/*) line 12 lignin tended to be lower than that of wild-type lignin (Supplementary Table 2).

Together, these data show that, although the growth of the *CCR2*(−/*) line 12 trees is similar to that of wild-type trees, their wood composition still reflects a deficiency in CCR2.

### Increased saccharification efficiency in *CCR2*(−/*) line 12

**Fig. 4:**
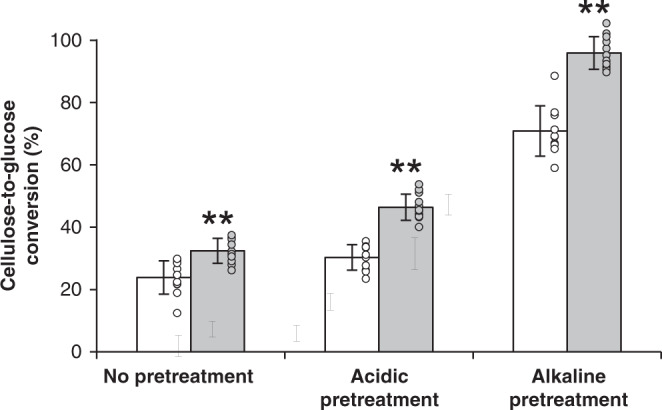
Saccharification assays of *CCR2*(−/*) line 12 stems. Plants were grown for 20 weeks in the greenhouse. Cellulose-to-glucose conversion efficiencies of wild type and *CCR2*(−/*) line 12 were calculated based on the quantified amounts of cellulose and the amount of released glucose after 72 h of saccharification following no pretreatment, acidic pretreatment (1 M HCl), or alkaline pretreatment (62.5 mM NaOH) (Supplementary Table 3, Table 1). Individual values (dots) and means (bars) of 10 biologically independent samples for wild type (white bars) and eleven biologically independent samples for *CCR2*(−/*) line 12 (gray bars). **P < 0.01 (two-tailed Student’s *t*-test); *P* = 5.1 × 10^−4^, 3.4 × 10^−8^, and 6.6 × 10^−8^ in the case of no pretreatment, acid pretreatment, and alkaline pretreatment, respectively (see also Supplementary Table 3). Error bars indicate standard deviation. Source data are provided as a Source data file.

Because lignin amount, composition, and polymerization degree greatly influence saccharification yield, we further investigated the saccharification potential of *CCR2*(−/*) line 12 under conditions of limited saccharification. The cellulose-to-glucose conversion was calculated based on the amount of glucose released upon saccharification of dried debarked stem material after either acidic (1 M HCl, 80 °C, 2 h), alkaline (62.5 mM NaOH, 90 °C, 3 h), or no pretreatment (Supplementary Table 3) and the original cellulose content that was measured for each sample (Table 1). In all three cases, cellulose-to-glucose conversion of biomass from *CCR2*(−/*) line 12 was significantly higher than that of the wild type (Fig. 4); the cellulose-to-glucose conversion of the non-pretreated samples increased from 23.9% in the wild type to 32.4% in *CCR2*(−/*) line 12 (i.e., a relative increase of 36%), after acidic pretreatment from 30.3 to 46.4% (i.e., a relative increase of 53%), and after alkaline pretreatment from 70.9 to 95.9% (i.e., a relative increase of 35%).

In many plant species, including Arabidopsis, tobacco, and poplar, lowering CCR activity leads to a substantial yield penalty^3,5,18,19^. As *CCR2*(−/*) line 12 had a biomass yield and cellulose amount that was comparable to that of the wild type, the saccharification yield expressed on a per plant basis was also increased in *CCR2*(−/*) line 12 under the used pretreatment conditions (Supplementary Table 3); using no pretreatment, acidic pretreatment, and alkaline pretreatment, the glucose yield per plant of *CCR2*(−/*) line 12 was increased by 25.3%, 41.3%, and 24.9%, respectively.

### Haplosufficient wild-type *P. tremula* × *P. alba CCR2* alleles

In *CCR2*(−/*) line 12, the *P. tremula CCR2* allele contained a frameshift mutation, thereby fully knocking out this allele. However, the effect of the 114I115A-to-114T amino-acid change in the protein encoded by the mutant *P. alba CCR2* allele on total CCR activity was not known. Either the 114I115A-to-114T amino acid change in the *P. alba* CCR2 protein did not have any effect on the CCR2 activity, in which case the red xylem phenotype and the reduced lignin amount of *CCR2*(−/*) line 12 can be explained by the haploinsufficiency of the wild-type *P. alba CCR2* allele. In this case, the CCR activity of the protein encoded solely by the wild-type *P. alba CCR2* allele does not suffice to secure normal lignin biosynthesis. Alternatively, the wild-type *P. alba CCR2* allele is haplosufficient, and the amino-acid change in the mutant *P. alba* CCR2 protein encoded by *CCR2*(−/*) line 12 reduces CCR2 activity resulting in the reduced lignin amount and the consequent red xylem phenotype.

**Fig. 5:**
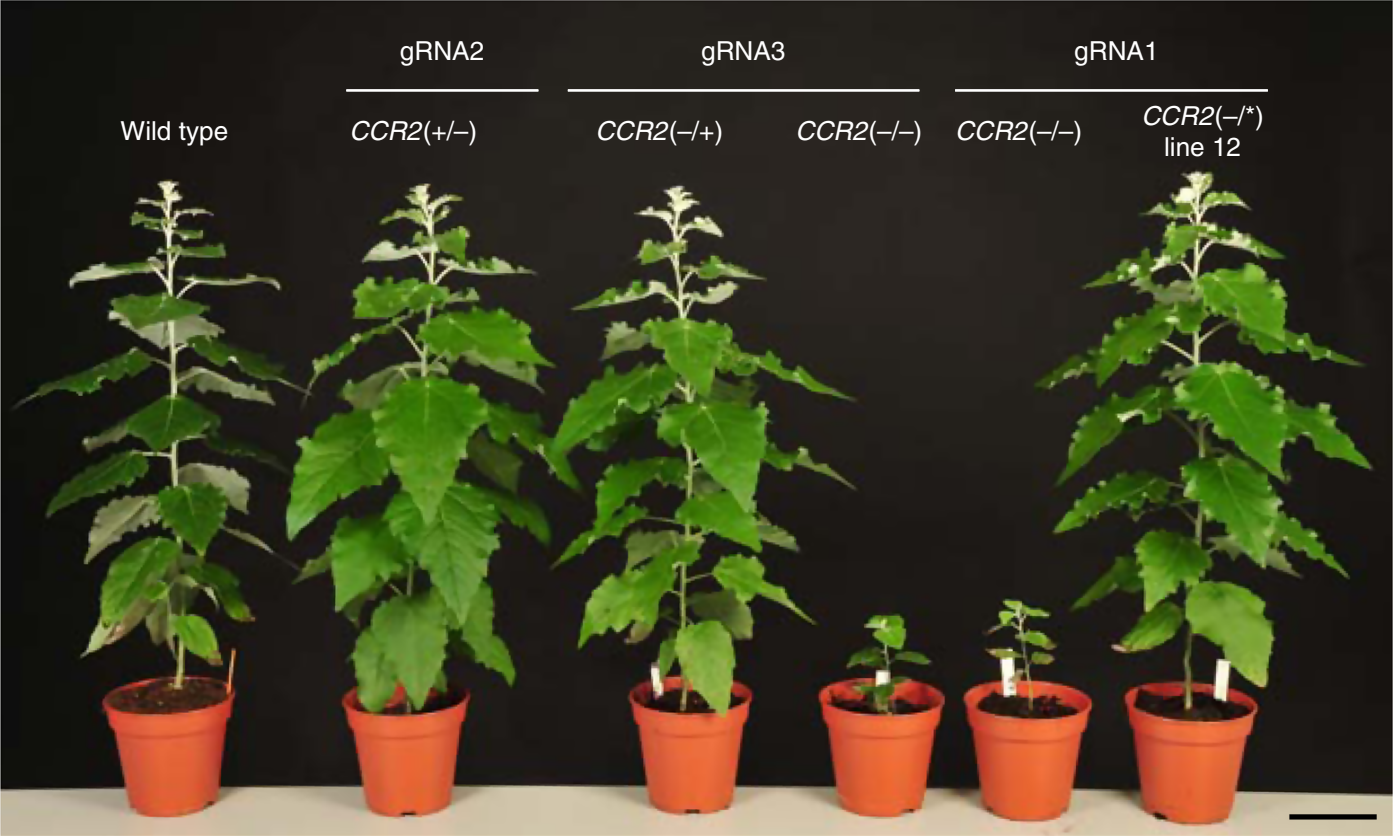
Phenotype of poplar containing mono- and biallelic *CCR2* mutations. Plants were grown for 11 weeks in the greenhouse. *CCR2*(+/−) generated via CRISPR/Cas9 using gRNA2, *CCR2*(−/+) and *CCR2*(−/−) generated via CRISPR/Cas9 using gRNA3, and *CCR2*(−/−) and *CCR2*(−/*) line 12 generated via CRISPR/Cas9 using gRNA1. gRNA1 targets the third exon of both *CCR2* alleles whereas gRNA2 and gRNA3 target the fourth exon of the *P. alba* or *P. tremula CCR2* allele, respectively. The status of the *CCR2* alleles present in *P. tremula* × *P. alba* is denoted between the parentheses; the first one represents that of the *P. tremula* allele, the second one that of the *P. alba* allele; +, wild type; −, knockout; *, protein-modified. The plants shown are representative for seven biologically independent samples for wild type, *CCR2*(+/−), *CCR2*(−/+), *CCR2*(−/−) (gRNA1), and *CCR2*(−/*) line 12. One plant was available for *CCR2*(−/−) (gRNA3). Scale bar = 10 cm.

To test whether a single *CCR2* allele, be it that encoded by the *P. tremula* or that by the *P. alba* genome, is sufficient to secure wild-type lignin amount, monoallelic null mutants in *CCR2* were generated by CRISPR/Cas9. gRNA2 and gRNA3 were used, targetting the fourth exon of the *CCR2 P. alba* and *P. tremula* allele, respectively (Supplementary Fig. 4a, 5a). Six lines contained monoallelic frameshift mutations in *P. alba CCR2* (*CCR2*(+/−); Supplementary Fig. 4b), while six out of eight lines in which the *P. tremula CCR2* allele was targeted contained monoallelic frameshift mutations in *P. tremula CCR2* (*CCR2*(−/+); Supplementary Fig. 5b). The other two lines contained biallelic mutations in *CCR2*. *CCR2*(−/−) line 202 contained biallelic frameshift mutations in *CCR2* and was severely dwarfed, in agreement with the biallelic frameshift mutants generated previously using gRNA1 (Supplementary Fig. 5b, Figs. 1 and 5). *CCR2*(−/*) line 206 contained a frameshift mutation (5-bp deletion) in the *P. tremula CCR2* allele, and a deletion of 3 bp in the *P. alba CCR2* allele (Supplementary Fig. 5b) and will be discussed further below.

**Fig. 6:**
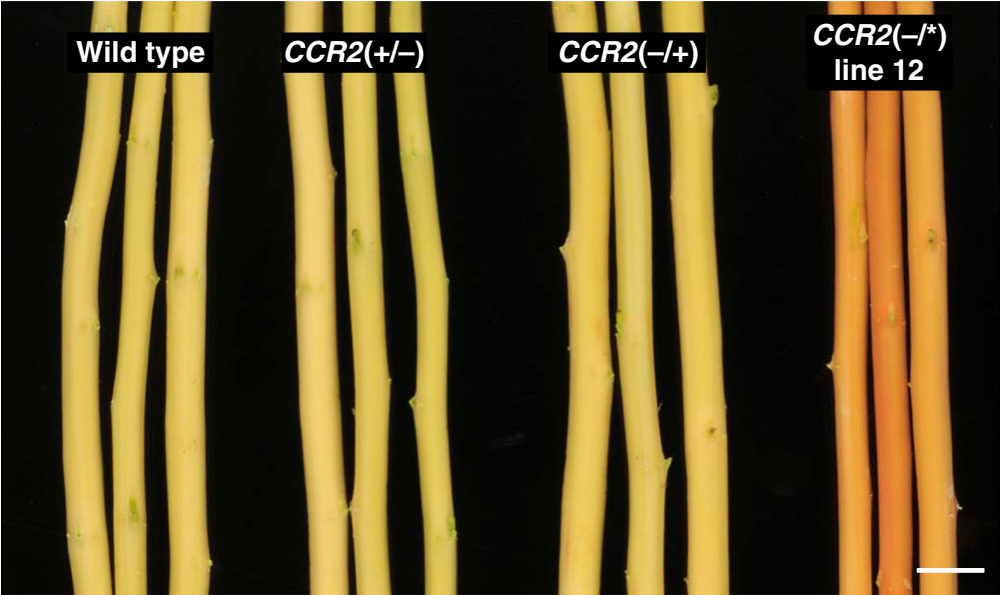
Phenotype of debarked stems of *CCR2*(+/−), *CCR2*(−/+), and *CCR2*(−/*) line 12 poplars. Plants were grown for 11 weeks in the greenhouse. The red xylem phenotype was only present in *CCR2*(−/*) line 12. The status of the *CCR2* alleles present in *P. tremula* × *P. alba* is denoted between the parentheses; the first one represents that of the *P. tremula* allele, the second one that of the *P. alba* allele; +, wild type; −, knockout; *, protein-modified. The stems shown are representative of seven biologically independent samples per line. Scale bar = 1 cm.

The twelve individual plants containing monoallelic frameshift mutations in *CCR2* (as shown in Supplementary Figs. 4b and 5b) were grown along with wild type and *CCR2*(−/*) line 12 in the greenhouse. After a growth period of 11 weeks, both the *CCR2*(+/−) and *CCR2*(−/+) monoallelic knock-outs and *CCR2*(−/*) line 12 plants were equal to the wild type in stem height and diameter, as well as fresh and dry weight (Table 2, Fig. 5). After debarking the harvested stems, the typical red coloration of the xylem was seen to be present in *CCR2*(−/*) line 12, but absent in the wild-type and the *CCR2* monoallelic knockout plants (Fig. 6). These observations indicate that the lignin of the *CCR2* monoallelic knockout plants resembles that of the wild type and not that of *CCR2*(−/*) line 12. To validate this, the lignin content and composition of dried debarked stem material were determined (Table 2). The acetyl bromide lignin content in the CWR from *CCR2* monoallelic knockout plants was equal to that of the wild type, whereas that of the *CCR2*(−/*) line 12 was reduced by ~15%. In agreement with the normal xylem coloration, no significant differences in thioacidolysis-released aromatic units were observed between the *CCR2* monoallelic knockout plants and the wild type. Similar to the results of 20-week-old stems, 11-week-old *CCR2*(−/*) line 12 stems had a decreased frequency of H monomers and an increased frequency of all three thioacidolysis-released ferulic acid units when compared to the wild-type and *CCR2* monoallelic knockout plants.

**Table 2:** Biomass and cell wall composition of *CCR2*(−/+), *CCR2*(+/−), and *CCR2*(−/*) line 12 stems.

	Wild type	*CCR2*(−/+)	*CCR2*(+/−)	*CCR2*(−/*) line 12
Fresh weight (g)	4.9 ± 0.5	3.4 ± 2.6 (0.276)	5.8 ± 1.6 (0.355)	4.0 ± 1.3 (0.640)
Dry weight (g)	1.1 ± 0.2	0.8 ± 0.6 (0.389)	1.4 ± 0.5 (0.530)	0.9 ± 0.3 (0.528)
Height (cm)	59.3 ± 2.7	51.8 ± 12.6 (0.237)	64.2 ± 7.5 (0.564)	53.0 ± 4.2 (0.331)
Diameter (mm)	6.0 ± 0.0	4.8 ± 1.2 (0.052)	6.4 ± 0.8 (0.674)	5.5 ± 0.6 (0.521)
CWR (% dry weight)	70.6 ± 4.3	75.6 ± 2.0 (0.174)	73.3 ± 2.6 (0.628)	74.4 ± 7.5 (0.334)
Acetyl bromide lignin amount (% CWR)	16.5 ± 1.2	16.2 ± 0.7 (0.888)	16.7 ± 0.7 (0.936)	14.0 ± 0.8** (<0.0001)
*Thioacidolysis-released monomers*				
H + G + S	34.1 ± 1.7	35.2 ± 2.6 (0.896)	33.9 ± 3.9 (0.999)	34.0 ± 4.6 (0.999)
%H	0.9 ± 0.3	0.5 ± 0.1 (0.050)	0.6 ± 0.3 (0.053)	0.3 ± 0.1** (<0.0001)
%S	56.1 ± 0.9	55.7 ± 0.6 (0.806)	55.7 ± 1.0 (0.782)	55.0 ± 1.0 (0.080)
%G	41.9 ± 1.2	42.7 ± 0.9 (0.421)	42.8 ± 0.9 (0.311)	43.3 ± 1.4 (0.071)
S/G	1.34 ± 0.06	1.31 ± 0.04 (0.507)	1.30 ± 0.05 (0.417)	1.27 ± 0.06 (0.063)
%β-*O*-4-FA-I	0.010 ± 0.001	0.013 ± 0.001 (0.994)	0.013 ± 0.004 (0.992)	0.086 ± 0.037** (<0.0001)
%β-*O*-4-FA-II	0.005 ± 0.001	0.005 ± 0.001 (0.943)	0.004 ± 0.002 (0.966)	0.033 ± 0.004** (<0.0001)
%*bis*-β-*O*-4-FA	0.064 ± 0.021	0.058 ± 0.018 (0.999)	0.045 ± 0.033 (0.992)	0.365 ± 0.288** (0.00354)
%G-aldehydes	1.1 ± 0.2	0.9 ± 0.4 (0.898)	0.8 ± 0.5 (0.379)	0.9 ± 0.4 (0.919)

Plants were grown for 11 weeks in the greenhouse. Stem diameter was determined 3 cm above soil level. At the time of harvest, the height, fresh weight (without bark and leaves), and diameter of the stem were measured. After drying the debarked stems for 5 days, the dry weight was determined. Cell wall residue (CWR) was determined after sequential extraction of dry debarked stem material. Lignin content was determined via the acetyl bromide method. Lignin composition was determined by thioacidolysis. The sum of H, G, and S is expressed in μmol per g acetyl bromide lignin. %H + %G + %S = 100; other units are expressed versus H + G + S. The data represent means ± standard deviation (for biomass measurements: wild type, *CCR2*(−/+) and *CCR2*(+/−), *n* = 6 biologically independent samples and *CCR2*(−/*) line 12, *CCR2*(−/+), *CCR2*(+/−), *n* = 7 biologically independent samples; for cell wall analysis: wild type and *CCR2*(−/*) line 12, *n* = 7 biologically independent samples, *CCR2*(−/+) and *CCR2*(+/−), *n* = 6 biologically independent samples). **P < 0.01, one-way ANOVA with Dunnett’s post hoc test; the exact *P* value (for the pairwise comparison with the wild type) is shown between parentheses in the table. The status of the *CCR2* alleles present in *P. tremula* × *P. alba* is denoted between the parentheses; the first one represents that of the *P. tremula* allele, the second one represents that of the *P. alba* allele; +, wild type; −, knockout; *, protein-modified. Source data are provided as a Source data file.

Judged by the normal growth, lignin amount and lignin composition of the *CCR2* monoallelic knockout plants, the *P. tremula* and the *P. alba CCR2* alleles appear to both be haplosufficient in *P. tremula* × *P. alba*.

### Phenolic profiling of the different *CCR2* mutants

**Fig. 7:**
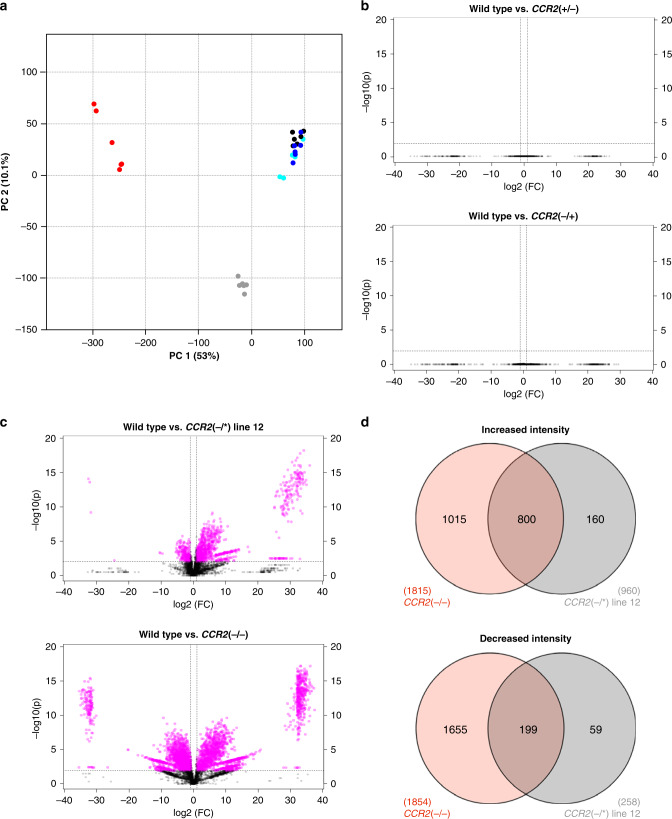
Phenolic profiling of *CCR2*(+/−), *CCR2*(−/+), *CCR*2(−/*) line 12, and *CCR2*(−/−) stems. Plants were grown for 11 weeks in the greenhouse. **a** Plot of principal component 1 (PC1) and PC2 of principal component analysis (PCA) on 6182 peaks. Black dots, wild type; blue dots, *CCR2*(+/−); cyan dots, *CCR2*(−/+); gray dots, *CCR2*(−/*) line 12; red dots, *CCR2*(−/−). **b**, **c** Volcano plots visualizing the differences between **b** wild type and *CCR2*(+/−) or *CCR2*(−/+), and **c** wild type and *CCR2*(−/*) line 12 or *CCR2*(−/−). Magenta and black dots represent peaks that are different and not different in intensity, respectively, based on the filters: fold change (FC) > 2 and *P* value <0.01 (one*-*way ANOVA with Dunnett’s post hoc test). **d** Venn diagrams of the number of peaks with significantly differential intensities (magenta dots from the volcano plots in (**c**)) between wild type and either *CCR2*(−/*) line 12 or *CCR2*(−/−). Wild type, *n* = 6 biologically independent samples; *CCR2*(−/*) line 12, *n* = 6 biologically independent samples; *CCR2*(−/−), *n* = 5 biologically independent samples.

To further examine the haplo(in)sufficient status of the *CCR2* alleles in *P. tremula *×* P. alba*, and investigate to what extent the metabolic changes of *CCR2*(−/*) line 12 reflect those expected from CCR2 deficiency, comparative phenolic profiling was performed via ultra-high-pressure liquid chromatography-mass spectrometry (UHPLC-MS) on debarked stems of wild type, *CCR2*(+/−), *CCR2*(−/+), *CCR2*(−/*) line 12, and *CCR2*(−/−). Principal component analysis (PCA) was performed on a total of 6182 peaks (mass-to-charge ratio [*m/z*] features) (Supplementary Data 1). PCA showed that, according to the first principal component (which explains 53% of the variation), the metabolic profiles of wild type, *CCR2*(+/−), and *CCR2*(−/+) were indistinguishable, whereas those of *CCR2*(−/*) line 12 were situated between those of *CCR2*(−/−) and wild type (Fig. 7a). The second principal component, which explains 10.1% of the variation, reflects variation within the genotypes (and not between the genotypes) and can be attributed to biological and/or technical variation. Next, univariate statistical analysis was applied to the selected peaks to screen for peaks with significantly different intensities in *CCR2*(+/−), *CCR2*(−/+), *CCR2*(−/*) line 12, or *CCR2*(−/−) mutants compared with their levels in wild-type plants. After applying specific filters (see “Methods”), no significant differences were found between either *CCR2*(+/−) or *CCR2*(−/+) and the wild type (Fig. 7b), again showing that the *P. tremula* and the *P. alba CCR2* alleles are both haplosufficient in *P. tremula* × *P. alba*. The reduction in lignin amount in *CCR2*(−/*) line 12 is therefore not solely a consequence of the *P. tremula CCR2* null allele.

By contrast, using the same specific filters, 960 and 1815 peaks were found to have a higher intensity in *CCR2*(−/*) line 12 and *CCR2*(−/−), respectively, when compared to the wild type, of which 800 were in common (Fig. 7c, d). In addition, 258 and 1854 peaks had a lower intensity in *CCR2*(−/*) line 12 and *CCR2*(−/−), respectively, when compared to the wild type, of which 199 were in common. Next, the top 10 highest peaks with significantly higher and lower intensities in *CCR2*(−/*) line 12 were structurally characterized based on their mass-to-charge ratio (*m/z*), retention time, and tandem mass spectrometry (MS/MS) (Supplementary Figs. 6–11, Supplementary Table 4). The ten highest peaks with significantly higher intensities in *CCR2*(−/*) line 12 could be assigned to ten compounds, of which seven could be (partially) structurally characterized as conjugates of ferulic acid, vanillic acid, sinapic acid, and caffeic acid, and three remained unknown. The ten highest peaks with significantly lower intensities in *CCR2*(−/*) line 12 could be assigned to nine compounds, of which five could be structurally characterized as oligolignols, and four remained unknown.

The relatively high fraction of differential peaks that *CCR2*(−/*) line 12 shares with *CCR2*(−/−) (Fig. 7d, Supplementary Table 4), and the identities of the differential compounds in *CCR2*(−/*) line 12, which are in line with the position of CCR2 in the lignin biosynthetic pathway (Supplementary Table 4), again underline the haploinsufficiency of the mutant *P. alba CCR2* allele in *CCR2*(−/*) line 12.

### A haploinsufficient *P. alba CCR2* allele in *CCR2*(−/*) line 12

In *CCR2*(−/*) line 12, the *P. alba* CCR2 protein sequence differs in only two amino acids from the wild-type *P. alba* CCR2 protein sequence (Supplementary Fig. 2). Because the analysis of monoallelic *CCR2* mutants suggested that the wild-type *CCR2* alleles are haplosufficient in *P. tremula* × *P. alba* (Table 2, Fig. 7), the reduced lignin content of *CCR2*(−/*) line 12 is probably the consequence of the mutation in the *P. alba CCR2* allele (in combination with the null *P. tremula CCR2* allele), which might encode an enzyme with a lower CCR activity or a lower protein stability as compared to the wild-type *P. alba CCR2*-encoded enzyme. To validate this, yeast assays were performed in which the activities of the wild-type and the mutan*t P. alba* CCR2 proteins were investigated based on the production of coniferaldehyde from feruloyl-CoA, the substrate of CCR2.

**Fig. 8:**
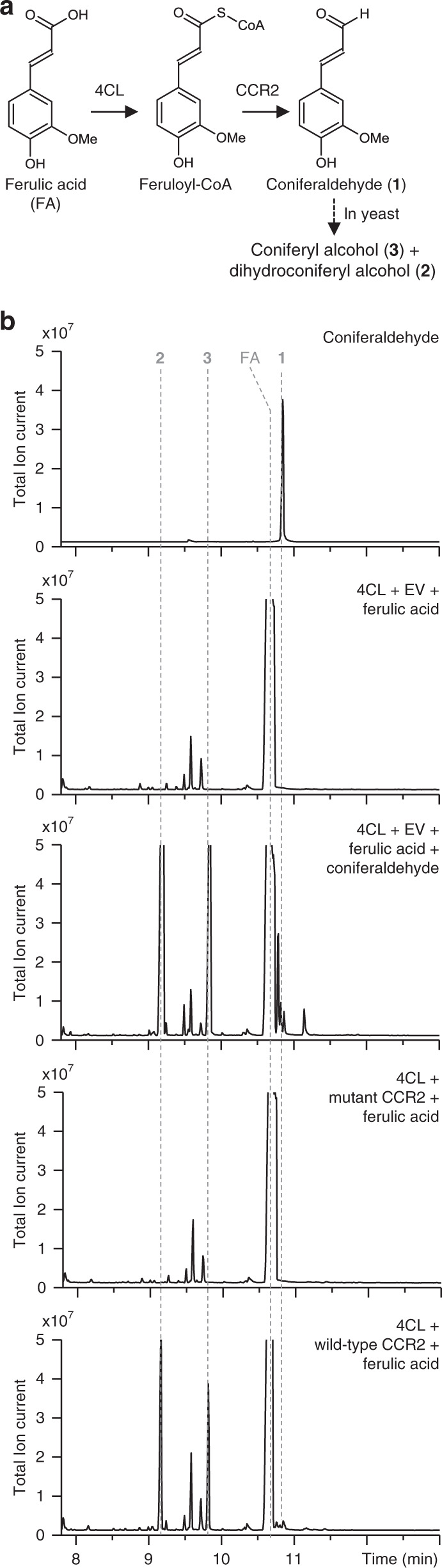
CCR2 activity assays in yeast. The relative activity of the mutant *P. alba* CCR2 protein (as present in *CCR2*(−/*) line 12) was determined in yeast. **a** Principle of the yeast feeding assay. Yeast cultures were engineered to express 4CL and the wild-type *P. alba* CCR2 protein or mutated *P. alba* CCR2 protein (as present in *CCR2*(−/*) line 12). After feeding the yeast cultures with ferulic acid, the activity of the respective CCR2 protein was judged based on the production of coniferaldehyde (the product of CCR2, peak 1), coniferyl alcohol (peak 2), and dihydroconiferyl alcohol (peak 3). See Supplementary Fig. 12 for the spectra of peaks 1–3. **b** GC-MS chromatograms of an authentic coniferaldehyde standard and extracts from ferulic acid-fed yeast cells expressing *4CL* in combination with either an empty vector (EV), mutant *P. alba CCR2* or wild-type *P. alba CCR2*. The results shown are representative of five biologically independent samples.

As it was not possible to feed feruloyl-CoA to the yeast cultures, whereas it was possible to feed ferulic acid, we created yeast strains that express *4-coumarate:CoA ligase* (*4CL*). The 4CL enzyme converts ferulic acid to feruloyl-CoA, allowing the CCR2 enzyme activities to be tested (Fig. 8a). All yeast cultures were fed with ferulic acid and extracts were analyzed by GC-MS. Yeast cultures expressing only *4CL* produced no coniferaldehyde upon feeding with ferulic acid (Fig. 8b). However, upon co-expression of *4CL* and wild-type *P. alba CCR2*, coniferaldehyde was formed (peak 1, Fig. 8b; Supplementary Fig. 12a, b). In addition to coniferaldehyde, two other metabolites, which were identified based on their EI-MS spectra as dihydroconiferyl alcohol and coniferyl alcohol (peak 2 and peak 3, respectively), accumulated in this strain (Fig. 8b; Supplementary Fig. 12c, d). As these two metabolites also accumulated in yeast cultures expressing only *4CL* and additionally fed with coniferaldehyde (Fig. 8b), we concluded that yeast cells further metabolize coniferaldehyde to dihydroconiferyl alcohol and coniferyl alcohol. Therefore, these two metabolites can be used as additional diagnostic markers for the production of coniferaldehyde in yeast cultures.

In contrast to yeast cultures expressing both *4CL* and the wild-type *CCR2*, yeast cultures expressing *4CL* and the mutated *P. alba CCR2* failed to produce coniferaldehyde, dihydroconiferyl alcohol, and coniferyl alcohol (Fig. 8b). Based on these data, we concluded that, in yeast, no detectable enzymatic activity was present for the mutant *P. alba* CCR2 protein. The normal growth but reduced lignin content of *CCR2*(−/*) line 12 suggests that, *in planta*, the mutant *P. alba* CCR2 protein had a reduced CCR activity and/or reduced protein stability as compared to the wild-type *P. alba* CCR2 protein, but did not fully lose its activity, as this would lead to dwarfism as observed in the *CCR2*(−/−) knockout mutants. The apparent null-activity in yeast might be explained by the lack of the proper cellular context of a normal lignifying cell, such as pH, interaction, and/or stabilization partners, etc., which might influence CCR2 enzymatic activity.

### Not all *CCR2*(−/*) lines display a normal growth phenotype

Using gRNA3 targeting the fourth exon of *CCR2*, another *CCR2*(−/*) line was obtained having an indel pattern in the *CCR2* alleles that was similar to that present in the *CCR2* alleles of *CCR2*(−/*) line 12 (albeit in a different position of the *CCR2* gene). *CCR2*(−/*) line 206 contained a frameshift mutation (5-bp deletion) in the *P. tremula CCR2* allele, and a deletion of 3 bp in the *P. alba CCR2* allele (Supplementary Fig. 5b). The latter resulted in a substitution of Trp175 and Asp176 for a Tyr175 in the corresponding *P. alba* CCR2 protein sequence (Supplementary Fig. 13). The amino-acid change occurred in α6 of the CCR2 protein, but not in the active site, NAPD-binding domain, or substrate-binding pocket residues^9–11^.

*CCR2*(−/*) line 206 had a lignin amount and growth that was increased when compared to that of *CCR2*(−/−), but still severely reduced when compared to that of the wild type (Supplementary Fig. 14a, b, d). However, similar to *CCR2*(−/−) stems, *CCR2*(−/*) line 206 stems also displayed a red xylem coloration and collapsed vessels (Supplementary Fig. 14c, d).

## Discussion

In previously generated *CCR2*-downregulated poplars, the red xylem phenotype appeared in patches, even among clones originating from the same plant, as a consequence of unstable downregulation^3^. A similar observation was made for poplars with RNAi-mediated downregulation of *4CL1*^4^. In contrast, *CCR2*(−/*) line 12 poplars had a stable reduction in lignin amount along the stem and in all biological replicates, as judged from the uniformly distributed red xylem phenotype, which is a big step forward in achieving stably modified lignocellulosic biomass.

After alkaline pretreatment, the cellulose-to-glucose conversion increased from 70.9% in wild type to an almost complete conversion (95.9%) in *CCR2*(−/*) line 12. Also without pretreatment, *CCR2*(−/*) line 12 had a 36% higher cellulose-to-glucose conversion than the wild type. Since no biomass penalty was observed for *CCR2*(−/*) line 12, even on a plant basis, this line yielded substantially more sugar than the wild type upon limited-saccharification experiments. However, before further translation of this knowledge to generate feedstock for the biorefinery, *CCR2*(−/*) line 12 poplars remain to be evaluated for growth and lignin amount when cultivated in the field, where they are exposed to different biotic and abiotic stresses. Indeed, it has been shown that genetically modified trees that grow normally in the greenhouse do not always grow as well as wild type when grown in the field^20^. But even if *CCR2*(−/*) line 12 plants would have a yield penalty in the field, the specific mutation present in the *P. alba CCR2* allele of *CCR2*(−/*) line 12 might still be valuable to engineer lignin in another cultivar or crop (such as Eucalyptus), because the effect of a mutation on the phenotype depends not only on the environment, but also on the specific genetic background in which it resides^21^.

Typically, using CRISPR/Cas9, knockout lines are generated. However, knockout mutants in lignin biosynthesis frequently suffer from undesired phenotypes such as dwarfism^6–8,18^. Lignin levels in plants can be reduced without affecting plant growth, but if lignin drops below a critical level, effects on vessel architecture and growth become apparent^7,8,18,22^. For example, for *CCR2*-downregulated (RNAi-mediated) poplars, a range of plants with different levels of *CCR2* downregulation and growth was obtained (even amongst biological replicates originating from the same plant)^3^. Approximately 5% of all *CCR2*-downregulated lines that have been generated displayed severe dwarfism and could only survive in tissue culture^5^. These lines were most likely the plants with the highest reduction in CCR2 activity. Of the *CCR2*-downregulated plants that survived on soil, the plants with the highest amount of red coloration (and thus the lowest amount of *CCR* expression and lignin amount) had the highest increase in saccharification efficiency, but also showed collapsed vessels and suffered from a biomass yield penalty^3,5^. Stems with lower amounts of red coloration did not suffer from a yield penalty, but only had a marginal reduction in lignin and increase in saccharification efficiency. Here, a similar observation was made, but in individual, stably CRISPR/Cas9-edited poplars; *CCR2*(−/−) knock-outs had the lowest amounts of lignin and displayed collapsed vessels and severe dwarfism. *CCR2*(−/*) line 206 had a slightly increased amount of lignin and biomass yield when compared to *CCR2*(−/−) but still displayed collapsed vessels, whereas *CCR2*(−/*) line 12, with its mild reduction in lignin amount compared to the wild type, displayed normal vessels and growth, even in clonally replicated trees. As the wild-type *CCR2* alleles are both haplosufficient in *P. tremula* × *P. alba*, the differences in lignin amount and growth between *CCR2*(−/*) line 206 and line 12 could be attributed to the 3 bp deletions occurring in the fourth and third exon of *P. alba CCR2* of *CCR2*(−/*) line 206 and line 12, respectively (see Supplementary Note 1 for an explanation of the impossibility of the off-target effect).

Our research shows that generating a range of allelic variants in the gene of interest by CRISPR/Cas9 is a useful strategy to pick up rare alleles that allow obtaining the desired (intermediate) phenotype (e.g., plants with reduced amounts of lignin without displaying a yield penalty, such as *CCR2*(−/*) line 12). For haplosufficient genes in outbreeding diploid species (such as most tree species), an interesting strategy is to knock out one allele, while the other can be screened for mutations leading to (small) amino-acid modifications, as described in this paper. Alternatively, screening for lines in which both alleles contain mutations leading to (small) amino-acid modifications might also be valuable. The yeast assay shows that, although it seems to be a fast, cheap, and easy way to evaluate the activity of specific allelic variants—or even to screen for specific allelic variants with modified activity—the results obtained are not necessarily good predictors for the activity of such variants *in planta*. Therefore, a direct *in planta* screening for such variants is a better strategy.

## Methods

### Plant material and vector construction

To introduce biallelic mutations in *CCR2*, a list of 30 protospacers with the N20-NGG motif specific for the *P. tremula* × *P. alba CCR2* alleles (Potri.003G181400) was extracted from the Aspen database (http://aspendb.uga.edu/)^23,24^. Next, the possible protospacers were analyzed based on their position in the *CCR2* alleles and the possible off-targets via the Aspen database^23,24^. In addition, GC-content and absence of a TTTTT sequence were considered. Based on these parameters, the most suitable gRNA sequence was chosen: GACCAAAAATGTGATCATTG (gRNA1). gRNA1 targets the third exon of both *CCR2* alleles. Using the *pUC gRNA Shuttle* (Addgene plasmid #47024) plasmid, gRNA1 was cloned into the *p201N:Cas9* plasmid (Addgene plasmid #59175) by Gibson assembly^25^. The *p201NCas9:gRNA1_CCR2* vector was transferred into *Agrobacterium tumefaciens* strain C58C1 pMP90 by electroporation. Agrobacterium-mediated transformation of *P. tremula* *×* *P. alba* 717-1B4 was performed via co-cultivation^26^. For this, 40 explants (which were preincubated on solidified M1 medium (see below) for 48 h at 24 °C in the dark) were dipped into 25 mL of Agrobacterium solution (grown on M liquid medium (see below) until reaching a concentration of 5 × 10^8^ cfu per mL) and slowly stirred for 16 h. After blotting on sterile paper, the explants were placed on solidified M1 medium for 48 h. Subsequently, the explants were washed with tetracycline solution (25 mg per liter) and sterile water, and transferred onto M2 medium (see below) for 10 days at 24 °C. Transferring the explants to solid M3 medium (see below) with 100 mg per liter kanamycin in standard light conditions yielded regenerated shoots that could be excised and transferred to M1/2 medium (see below) with 50 mg per liter kanamycin. After growing for approximately 20 days in standard light conditions, the plants (that developed roots and elongated shoots) were micropropagated on M1/2 medium (see below) with 50 mg per liter kanamycin.

To introduce monoallelic mutations in *CCR2*, specific gRNAs (targeting either the *P. tremula* or the *P. alba CCR2* allele) were selected based on the criteria described above. The best suitable gRNA sequences were GTGGTATTGCTATGGAAAGG (gRNA2) and GGAACAAGCTGCATGGGATA (gRNA3). gRNA2 and gRNA3 target the fourth exon of the *P. alba* and *P. tremula CCR2* allele, respectively. Cloning of the gRNAs in the *p201N-Cas9* vector, subsequent transformation into *A. tumefaciens* and poplar transformation were performed as described above.

For genotyping the regenerated, transformed shoots, the following primers pairs (designed using the Primer3 0.4.0 software) were used for PCR for identifying the mutations present in the *CCR2* alleles. For the PCR using the DNA extracted from plants transformed with the construct containing gRNA1: forward 5′-TACAYGGTAATTAATGGTGG-3′, reverse 5′-GATACCTTGGTGTTCTTGC-3′; for gRNA2: forward 5′-AGCTTGCCCGTTCTGTGTT-3′, reverse 5′-CGGTGAGGTACTTGAGGATG-3′; for gRNA3: forward 5′-ACCCCGTTCTGGTAGCTG-3′, reverse 5′-GGAAGGCGTCTCAAAGACT-3′. The PCR products were sequenced by Eurofins (Eurofins Genomics) and analyzed via CLC Main Workbench 8.

The wild-type controls (*P. tremula* *×* *P. alba* 717-1B4) originate from the same batch of plants that was used to generate the transgenic lines. However, instead of generating callus-tissue and performing Agrobacterium-mediated transformation, the wild-type controls were maintained and clonally propagated to serve as a control for the regenerated, transformed shoots.

For clonal propagation, poplars that were grown for 3 to 4 months in tissue culture (on M1/2 medium as described below) under long-day conditions (16-h light and 8-h dark photoperiod, 24 °C) were used. More specifically, the stems were cut into pieces of ±2 cm and put on fresh M1/2 medium to allow the development of roots and new shoots.

### Media used for plant cultivation

M: Murashige and Skoog basic medium (Duchefa) 4.4 g per liter, Morel and Wetmore vitamins 10 mL per liter, *L*-cystein 1 mg per liter, *L*-glutamine 200 mg per liter, sucrose 30 g per liter, plant agar (Duchefa) 6.2 g per liter.

M1/2: as M but with half-strength macro-nutrients, sucrose 20 g per liter, IAA 3 µM.

M1: as M but with NAA 10 µM and 2iP 5 µM.

M2: as M1 but with carbenicillin 500 mg per liter, cefotaxime 250 mg per liter.

M3: as M but with carbenicillin 500 mg per liter, cefotaxime 250 mg per liter, thidiazuron 0.1 µM.

### Plant growth and harvest

After growing for four months in tissue culture under long-day conditions, the transgenic poplars and their wild-type controls were transferred to soil. More specifically, the poplars were transferred to pots of 5.5-cm diameter filled with Saniflor commercial soil (Van Israel nv), placed in a tray filled with water, and covered with a cage liner (Tecniplast APET disposable cage liner for cage body 1291H) for acclimatization. After 2 weeks, one side of the cage liner was lifted 1 cm above water level and kept accordingly for 1 day, after which the other side also was lifted. The next day, the cage liner was removed and the acclimatized plants were transferred to bigger pots filled with a Saniflor commercial soil (Van Israel nv) (10 liter). Note that *CCR2*(−/−) plants were found to be extremely sensitive to the adjustment to greenhouse conditions. Therefore, here, the cage liner was kept closed for a period of 5 weeks and slowly opened over a period of 3 weeks to allow a much slower transition to the less-humid greenhouse conditions. Even with this precautionary measure, only a small number of *CCR2*(−/−) poplars recovered, whereas all plants of the other genotypes survived. In total, six batches of plants were grown for 11 or 20 weeks in the greenhouse under a 16-h light and 8-h dark photoperiod at ~21 °C.

The first batch, containing wild type, the 20 individual *CCR2*(−/−) lines, and *CCR2*(−/*) line 12, was grown for 11 weeks in the greenhouse and used for a picture of the growth phenotype (Fig. 1).

The second batch, containing wild type and *CCR2*(−/*) line 12, was grown for 20 weeks in the greenhouse. The height of the trees was determined weekly (Fig. 2a, b). After that, the diameter of the stems was determined (3 cm above soil level) (Table 1). Next, the stems were harvested (40 cm above soil level), the leaves were removed from the stem and the fresh weight of the stems was determined before and after removing the bark (Table 1). The dry weight of the debarked stems was determined after air-drying the stems for 2 weeks at ambient temperature (Table 1). The dried, debarked stem was ground in a ball mill for cell wall analysis and saccharification assays (Table 1, Fig. 4, Supplementary Tables 2 and 3).

The third batch consisted of the same lines present in batch 2 (wild type and *CCR2*(−/*) line 12), but now also accompanied by *CCR2*(−/−) mutants. These plants were also grown for 20 weeks in the greenhouse. For wild type and *CCR2*(−/*) line 12, 5-cm-long stem parts between 15 and 20 cm (relative to the soil) were harvested, debarked, and stored in tap water for 1 day. For *CCR2*(−/−), the 3-cm-long stem parts between 3 and 6 cm (relative to the soil) were harvested, debarked, and stored in tap water for 1 day. These stem pieces were used for microscopy (Fig. 3).

The fourth batch, containing wild type, *CCR2*(+/−), *CCR2*(−/+), and *CCR2*(−/*) line 12, was grown for 11 weeks in the greenhouse. After that, the diameter of the stems was determined (3 cm above soil level). Next, the stems were harvested (5 cm above soil level), and the fresh weight was determined after removing the leaves and bark. The dry weight was determined after drying the stems for 5 days at 50 °C (Table 2). For acetyl bromide and thioacidolysis (Table 2), the harvested stems were debarked, air-dried, and ground in a ball mill (Fig. 6).

The fifth batch consisted of the same lines present in batch 4 (wild type, *CCR2*(+/−), *CCR2*(−/+), and *CCR2*(−/*) line 12), but now also accompanied by *CCR2*(−/−) mutants (Fig. 5). After growing for 11 weeks in the greenhouse, the stems were harvested (5 cm above soil level) and the bottom 10 cm of the harvested stem piece was debarked, snap-frozen in liquid nitrogen, and stored at −70 °C for metabolomics (Fig. 7, Supplementary Table 4, Supplementary Figs. 6–11).

The sixth batch, containing wild type, *CCR2*(−/*) line 206, and *CCR2*(−/−) was grown for 20 weeks in the greenhouse. The height of the trees was determined weekly (Supplementary Fig. 14a–c). For the wild type, *CCR2*(−/*) line 206, and *CCR2*(−/−), the stems were harvested 10, 3, and 3 cm above soil level, respectively. Of each harvested wild-type, *CCR2*(−/*) line 206, and *CCR2*(−/−) stem pieces, the basal 3, 1, and 1 cm, respectively, was debarked and stored for 3 days in tap water for microscopy (Supplementary Fig. 14d).

### Microscopy

Fifteen-micrometer-thick stem slices were made using a Reichert-Jung 2040 Autocut Microtome (Leica). Sections were stained with Wiesner and Mäule reagents; Wiesner staining was performed by adding a drop of phloroglucinol-HCl solution (consisting of one volume of concentrated HCl (37 M) and two volumes of 3% phloroglucinol in ethanol) to the sections. Mäule staining was performed by incubating the sections in 0.5% (w/v) potassium permanganate for 5 min, followed by washing with distilled water. Next, the sections were incubated in concentrated HCl (37 M), followed by the addition of concentrated ammonium hydroxide solution.

Images were acquired using an Olympus BX51 microscope (Olympus) with an Olympus PlanC N 10x (0.25 NA) objective or Olympus UplanFl N 20x (0.50 NA) objective and the Toupview x64,3.7.10121 software. Sections were also imaged via autofluorescence using a Zeiss Axio Imager.M1 microscope (Carl Zeiss) with a Plan-Apochromat 10X (0.45 NA) objective and the AxioVs40 V4.8.2.0 software. A 365-nm excitation filter was used together with a 395-nm beamsplitter and a 420- to 470-nm bandpass emission filter.

### Cell wall characterization

To determine the matrix polysaccharides, cellulose, and lignin characteristics, ground wood powder was used for preparing cell wall residue by sequentially washing for 30 min each with milliQ water at 98 °C, ethanol at 76 °C, chloroform at 59 °C, and acetone at 54 °C. The remaining CWR was dried under vacuum.

To determine the crystalline cellulose amount, the Updegraff method^27^ was used on 10 mg of CWR. First, the samples were incubated with 1 mL trifluoroacetic acid (2 M) for 2 h at 99 °C while shaking at 750 rpm, followed by centrifugation for 3 min at 19,757 × *g*. Next, the pellet was washed three times with 1 mL milliQ water, two times with 1 mL acetone, dried under vacuum, and weighed. The weight loss upon trifluoroacetic acid digestion was used to determine the matrix polysaccharide content (including hemicelluloses, pectins, and amorphous cellulose). Subsequently, 1 mL of Updegraff reagent (consisting of eight volumes of acetic acid, one volume of nitric acid, and 2 volumes of milliQ water) was added to the pellet, followed by vortexing and heating at 99 °C for 30 min. After centrifuging the samples for 15 min at 10,080 × *g*, the supernatant was discarded without disturbing the pellet. Next, the pellet was washed one time with 1 mL water and two times with 1 mL acetone. After drying under vacuum, the pellet was incubated with 175 µL of 72% (v/v) sulfuric acid for 30 min at room temperature. Next, the samples were vortexed and incubated for another 15 min at room temperature. After adding 825 µL of water, the samples were centrifuged for 5 min at 10,080 × *g*. To wells of a 96-well plate, 5 µL of supernatant, 95 µL of water and 200 µL of freshly prepared anthrone reagent (2 mg anthrone per mL pure sulfuric acid) was added. The plate was sealed and incubated for 30 min at 80 °C. After cooling down to room temperature, the absorption was measured at 625 nm with a NanoDrop® ND-1000 spectrophotometer (Thermo Scientific). The Nanodrop 1000 3.8.1 software was used for collecting the absorption data.

Lignin content was determined by the acetyl bromide protocol^18,28^ on 5 mg of CWR, and the Klason protocol^7,29^ on 100 mg of CWR. In the acetyl bromide protocol, the samples were incubated in 250 µL freshly made acetyl bromide solution (25% acetyl bromide in glacial acetic acid) for 2 h at 50 °C. Next, the samples were incubated for another hour at 50 °C with vortexing every 15 min. After cooling the samples on ice and centrifuging for 15 min at 10,080 × *g*, 100 µL supernatant, 75 µL freshly made hydroxylamine hydrochloride (0.5 M), 400 µL NaOH (2 M), and 1.425 mL glacial acetic acid were added to an empty 2-mL Eppendorf. The lignin concentrations were determined by measuring the absorbance at 280 nm with a NanoDrop® ND-1000 spectrophotometer (Thermo Scientific) and applying the Bouguer–Lambert–Beer law. The Nanodrop 1000 3.8.1 software was used for collecting the absorption data. In the Klason protocol, the samples were incubated with 1 mL sulfuric acid (72%) in 15-mL glass vials for 2 h at room temperature while stirring. After transferring the samples to a 100-mL flask, 13.4 mL of milliQ water was added. The flasks were autoclaved for 1 h (1 bar, 121 °C). Next, the samples were incubated for 16 h at 4 °C. To measure the acid-soluble lignin, 1 mL of supernatant was collected (see further). The remaining samples were filtered and washed extensively with 200 mL of milliQ water through a preweighed filter paper (Sartorius AG) using a Büchner filter system (Merck Millipore). The filter papers were dried for 16 h at 105 °C and cooled down for 1 h at room temperature. The lignin amount was determined gravimetrically. For determining the acid-soluble lignin, the previously collected 1 mL of supernatant was centrifuged and diluted 20 times in milliQ water. The absorbance at 205 nm was measured using a spectrophotometer (Genesys 10 S UV-Vis, Thermo Scientific). The acid-soluble lignin concentrations were calculated by means of the Bouguer–Lambert–Beer law.

For the lignin composition determination via thioacidolysis^14,30^, 15 mg of CWR was weighed into a 5‐mL glass Wheaton vial with Teflon‐lined screw‐cap. First, the samples were incubated with 1 mL reaction mixture (consisting of 2.5% boron trifluoride etherate and 10% ethanethiol in dioxane) for 4 h at 98 °C, while shaking the vials manually every half an hour. Next, the samples were incubated for 5 min at −20 °C. To the samples, 0.2 mL tetracosane in dichloromethane (5 mg per mL) and 0.3 mL of 0.4 M sodium bicarbonate was added. Next, the samples were extracted by adding 2 mL milliQ water and 1 mL dichloromethane. By using a Pasteur pipette packed with a small cotton plug and a spatula-point of anhydrous sodium sulfate, 0.5 mL of the organic phase was filtered and added to a new Eppendorf. The samples were dried under vacuum and resuspended in 0.2 mL dichloromethane. Derivatisation occurred by adding 20 μL pyridine and 100 μL *N*,*O*‐bis(trimethylsilyl) acetamide to 20 μL of resuspended sample and incubating the samples for 2 h at 25 °C while shaking at 750 rpm. The reaction product was analyzed by GC-MS. GC-MS analysis was carried out using a 7890B GC system equipped with a 7693A Automatic Liquid Sampler and a 7250 Accurate-Mass Quadrupole Time-of-Flight MS system (Agilent Technologies). One microliter of the reaction product was injected in splitless mode with the injector port set to 280 °C. Separation was realized with a VF-5ms column (30 m × 0.25 mm, 0.25 μm; Varian CP9013; Agilent Technologies) with helium carrier gas at a constant flow of 1.2 mL per min. The oven was held at 130 °C for 3 min post-injection, ramped to 200 °C at 10 °C per min, ramped to 250 °C at 3 °C per min, held at 250 °C for 5 min, ramped to 320 °C at 20 °C per min, held at 320 °C for 5 min, and finally cooled to 130 °C at 50 °C per min at the end of the run. The MSD transfer line was set to 280 °C and the electron ionization energy was 70 eV. Full EI-MS spectra were recorded between *m*/*z* 50 and 800 at a resolution of >25,000 and with a solvent delay of 10.0 min. Peak integrations for quantification of the lignin monomers were carried out using the MassHunter Quantitative Analysis (for QTOF) software package (Agilent Technologies).

For the lignin composition determination via NMR^31–33^, 40–50 mg of ground stem powder was suspended in 0.6 mL DMSO-d_6_:pyridine-d_5_ (4:1, v/v). The samples were sonicated, with occasional mixing by vortexing, until a uniform gel was formed. NMR experiments were performed on a Bruker Biospin (Billerica) Avance 700 MHz spectrometer equipped with a 5-mm QCI ^1^H/^31^P/^13^C/^15^N cryoprobe with inverse geometry (proton coils closest to the sample). As in internal reference, the central DMSO solvent peak was used (δ_C_ 39.5, δ_H_ 2.49 ppm). The ^1^H–^13^C correlation experiment was an adiabatic HSQC experiment (Bruker standard pulse sequence ‘hsqcetgpsisp2.2’; phase-sensitive gradient-edited-2D HSQC using adiabatic pulses for inversion and refocusing). The parameters used for the HSQC experiments were acquired from 10 to 0 ppm in F2 (^1^H) with 1398 data points (acquisition time, 100 ms) and 200 to 0 ppm in F1 (^13^C) with 570 increments (F1 acquisition time, 8 ms) of 32 scans with a 1 s interscan delay. The d_24_ delay was set to 0.86 ms (1/8 J, J = 145 Hz). The total acquisition time for a sample was ~5 h. Processing used typical matched Gaussian apodization (GB = 0.001, LB = −0.5) in F2 and Gaussian apodization (GB = 0.001, LB = −0.3) also in F1 (without using linear prediction). Volume integration of contours in HSQC plots used TopSpin 4.0.8 software, and no correction factors were used.

### Analytical gel-permeation chromatography

Ground powder (~150 mg) was treated with cellulase (5 wt% to biomass) in sodium acetate buffer (pH 5.0, 40 mL) at 37 °C for 72 h. After centrifugation, the precipitate was collected and washed five times with deionized water to obtain enzyme lignin for gel-permeation chromatography (GPC) analysis.

Enzyme lignin (~10 mg) was dissolved in dimethylformamide (DMF)/lithium bromide (LiBr) (700 μL, 0.1 M LiBr) solution and filtered through a syringe filter (0.45 μm, PVDF). GPC analysis was performed utilizing a Shimadzu LC20-AD LC pump equipped with a Shimadzu SPD-M20A UV-vis detector set at 280 nm and a Shimadzu RID-10A refractive index detector. The GPC column set consists of 4 TOSOH (TOSOH Bioscience, LLC) GPC columns and a guard column (TSKgel Guard Alpha 6.0 mm ID × 4.0 cm, 13 μm → TSKgel Alpha-M 7.8 mm ID × 30 cm, 13 μm → TSKgel Alpha-M 7.8 mm ID × 30 cm, 13 μm → TSKgel Alpha-2500 7.8 mm ID × 30 cm, 7 μm → TSKgel Alpha-2500 7.8 mm ID × 30 cm, 7 μm). The column oven was held at 50 °C during analysis. The mobile phase was DMF with 0.1 M LiBr, the flow rate was 0.5 mL per min, and the oven temperature was 40 °C on an injection volume of 10 μL. Molecular weight distributions were determined using Shimadzu GPC postrun software via a conventional calibration curve using a ReadyCal polystyrene Kit (Sigma-Aldrich, Aldrich # 76552, M(p) 250-70000).

### Saccharification assay

Saccharification was performed on 10 mg of dried, ground stem material^34^. The samples were saccharified for 72 h using no pretreatment, acidic pretreatment (1 M HCl, 80 °C for 2 h while shaking at 750 rpm), or alkaline pretreatment (62.5 mM NaOH, 90 °C for 3 h while shaking at 750 rpm). After the pretreatment, the samples were centrifuged for 5 min at 10,080 × *g*. The pellet was washed three times with 1 mL milliQ water, and incubated in 1 mL of 70% ethanol for 16 h at 55 °C. After another centrifugation step (5 min at 10,080 × *g*), the pellet was washed three times with 1 mL 70% ethanol and one time with 1 mL acetone, centrifuged for 5 min at 10,080 × *g*, 10,000 rpm, dried under vacuum, and weighed.

The enzyme mix consisted of a 5:3 ratio of cellulase from *Trichoderma reseei* ATCC 26921 and β-glucosidase (Novozyme) which were first desalted over an EconoPac 10DG column (Bio-Rad), stacked with Bio-gel® P-6DG gel (Bio-Rad) according to the manufacturer’s guidelines. The activity of the enzyme mix was measured with a filter paper assay and was 0.18 FPU (filter paper units) per mL. Subsequently, the samples were dissolved in 1 mL acetic acid buffer solution (pH 4.8) and incubated at 50 °C at 750 rpm. After 5 min of incubation, 100 μL freshly prepared enzyme mix was added. After spinning down the samples in a benchtop microcentrifuge, 20 μL of the supernatant was taken after 72 h of incubation at 50 °C and 30-fold diluted with acetic acid buffer (pH 4.8). To determine the concentration of glucose in these samples, a spectrophotometric color reaction was used (glucose oxidase-peroxidase (GOD-POD); 100 mL of GOD-POD reaction mixture contained 50 mg 2,2′-azino-bis(3-ethylbenzthiazoline-6-sulfonic acid), 44.83 mg GOD, and 173 μL POD (4% (w/v)) in acetic acid buffer (pH 4.5)). To this end, 50 μL of 30-fold diluted sample was added to 150 μL GOD-POD mixture and incubated for 30 min at 37 °C. The absorbance was measured spectrophotometrically at a wavelength of 405 nm (Microplate-reader SpectraMax 250 (Sopachem), SoftMax Pro version 5 was used for collecting data). The concentration in the original sample was calculated with a standard curve based on known D-glucose concentrations.

### Phenolic profiling

For phenolic profiling, the frozen debarked stem parts were cut into little pieces using scissors. Subsequently, the stem pieces were extracted by adding 1 mL methanol and incubating for 15 min at 70 °C while shaking at 1000 rpm. After centrifugation at 19,757 × *g*, 800 μL of the supernatant was transferred to a new Eppendorf and dried under vacuum. After dissolving the pellet in 100 μL cyclohexane, 100 μL milliQ water was added and the cyclohexane/water mixture was vortexed. After centrifugation at 19,757 × *g*, 70 μL of the water phase was subjected to UHPLC-MS on an ACQUITY UPLC I-Class system (Waters) consisting of a binary pump, a vacuum degasser, an autosampler, and a column oven. Chromatographic separation was performed on an ACQUITY UPLC BEH C18 (150 × 2.1 mm, 1.7 μm) column (Waters), while maintaining the temperature at 40 °C. A gradient of two buffers (A and B) was utilized: buffer A (99:1:0.1 water:acetonitrile:formic acid, pH 3) and buffer B (99:1:0.1 acetonitrile:water:formic acid, pH 3), as follows: 99% A for 0.1 min decreased to 50% A in 30 min, decreased to 0% from 30 to 40 min. The flow rate was 0.35 mL per min, and the injection volume was 10 μL. This UHPLC system was connected to a Vion IMS QTOF hybrid mass spectrometer (Waters). The LockSpray ion source was used in negative electrospray ionization mode under the following specific conditions: capillary voltage, 3 kV; reference capillary voltage, 2.5 kV; cone voltage, 30 V; source offset, 50 V; source temperature, 120 °C; desolvation gas temperature, 550 °C; desolvation gas flow, 800 liter per h; and cone gas flow, 50 liter per h. The collision energy for full MSe was set at 6 eV (low energy) and ramped from 20 to 70 eV (high energy), intelligent data capture intensity threshold was set at 5. For DDA-MSMS, the low mass ramp was ramped between 15 and 30 eV. The high mass ramp was ramped between 30 and 70 eV. Nitrogen (greater than 99.5%) was used as desolvation and cone gas. Leucin-enkephalin (250 pg per μL solubilized in water:acetonitrile 1:1 (v/v), with 0.1% formic acid) was utilized for the lock mass calibration, with scanning every 2 min at a scan time of 0.1 s. Profile data were recorded through a UNIFI Scientific Information System (Waters). Data processing was performed with Progenesis QI software version 2.4 (Waters). All 30,662 peaks (mass-to-charge ratio [*m/z*] features) were integrated in the chromatograms of wild type, *CCR2*(+/−), *CCR2*(−/+), *CCR2*(−/*) line 12, and *CCR2*(−/−). The 6182 peaks that had an abundance of at least 0.01% of the highest average in the group with the highest peak abundance were selected for further analysis. After ANOVA, peaks with a *P* value < 0.01 (false discovery rate adjusted) and a twofold difference in abundance between mutant and the wild type were considered as being significantly different. PCA and volcano plots were generated using MetaboAnalyst 4.0. Chemdraw 16 was used for calculating exact *m/z* values.

### CCR2 activity assays in yeast

*Malus domestica 4CL* (*Md4CL*; GenBank Accession number XM_008365460) and yeast codon-optimized versions of the wild-type and mutant *P. alba CCR2* alleles (Supplementary Fig. 15) were Gateway-cloned into the donor vector pDONR207 and sequence verified. For expression in yeast, the *Md4CL* entry clone was Gateway-recombined into the high-copy number yeast destination vector pAG426GAL-ccdB (AddGene plasmid 1415535^35^). The wild-type and mutant *CCR2* alleles were also Gateway-recombined into the high-copy number yeast destination vector pAG424GAL-ccdB (AddGene plasmid 1415135^35^).

The resulting expression clones were used to transform yeast strain W303-1A (*MATa*; *leu2-3,112*, *trp1-1*, *can1-100*, *ura3-1*, *ade2-1*, *his3-11,15*) using the lithium acetate/single-stranded carrier DNA/polyethylene glycol method^36^. To this end, 50 mL YPD medium was inoculated with an overnight grown yeast pre-culture to an absorbance of 0.25 at 600 nm. Subsequently, the culture was grown until the absorbance reached 1.0 by incubating at 30 °C. After harvesting the cells by centrifugation, the cells were washed with 1 mL lithium acetate (0.1 M) and dissolved in 350 μL lithium acetate (0.1 M). For transformation, 50 μL-aliquots of the cells were transferred to an Eppendorf. To the aliquots, 200 μL PLI solution (for 50 mL PLI solution, mix 40 mL of 50% PEG with 5 mL lithium acetate (1 M) and 5 mL milliQ water), 10 μL salmon sperm ssDNA and plasmid DNA (1 μg of each plasmid) was added, followed by incubation for 30 min at 42 °C. After harvesting the cells by centrifugation, they were washed with 800 μL milliQ water and plated on selective plates with synthetic-defined (SD) medium (Clontech) supplemented with –Trp/–Ura amino-acid dropout supplement (Clontech). After incubating the plates for 2 days at 30 °C, five independent transformed colonies were selected. In total, five independent biological cultures were made for strains containing (1) pAG426GAL-*Md4CL* + pAG424GAL-ccdB (empty vector control), (2) pAG426GAL-*Md4CL* + pAG424GAL-wild type_*CCR2*, and (3) pAG426GAL-*Md4CL* + pAG424GAL-mutant_*CCR2*.

Yeast precultures of each of the five biological replicates were grown in SD medium with –Trp/–Ura dropout supplement (Clontech) for 24 h at 30 °C with shaking at 300 rpm. Gene expression was induced by washing the precultures with milliQ water and inoculating them in 10 mL SD Gal/Raf medium with –Trp/–Ura dropout supplement. After incubating the cultures for 24 h, 500 μL ferulic acid (20 mM, in 50:50 ethanol:water) or 500 μL coniferaldehyde (20 mM, in 50:50 ethanol:water) was added. The cultures were incubated further for 48 h. Finally, 1 mL of each yeast culture was harvested. After extracting the medium three times with 500 µL ethyl acetate, the solvent of the combined organic fractions was dried under vacuum. The remaining pellet was derivatized by using 10 µL pyridine and 50 µL *N*-methyl-*N*-trimethylsilyl-trifluoroacetamide (MSTFA) for GC-MS analysis on a GC model 6890 and MS model 5973 (Agilent Technologies). One microliter of the sample was injected in splitless mode with the injector port set to 280 °C. Separation was realized with a VF-5ms column (30 m × 0.25 mm, 0.25 μm; Varian CP9013; Agilent Technologies) with helium carrier gas at a constant flow of 1 mL per min. The oven was held at 80 °C for 1 min post-injection, ramped to 280 °C at 20 °C per min, held at 280 °C for 45 min, ramped to 320 °C at 20 °C per min, held at 320 °C for 1 min, and finally cooled to 80 °C at 50 °C per min at the end of the run. The MSD transfer line was set to 250 °C and the electron ionization energy was 70 eV. Full EI-MS spectra were recorded between *m*/*z* 60 and 800 with a solvent delay of 7.8 min. GC-MS data were recorded and visualized with Agilent firmware and visualized via the AMDIS software (Version 2.6, NIST).

### Statistical analyses

MS Excel 2016 and SAS 9.4 were used for statistical analysis. The specific method used is mentioned in the respective Table and Figure legends.

### Reporting summary

Further information on research design is available in the Nature Research Reporting Summary linked to this article.

## Supplementary information

Supplementary Information

Peer Review

Reporting summary

Description of Additional Supplementary Files

Supplementary Dataset 1

## Data Availability

Data supporting the findings of this work are available within the paper and its Supplementary Information files. A reporting summary for this Article is available as a Supplementary Information file. The datasets generated and analyzed during the current study are available from the corresponding author upon request. Sequences data that support the findings of this study were obtained from the Aspen database (for the *CCR2* sequences from *P. tremula* *×* *P. alba;* http://aspendb.uga.edu/) and NCBI (for the *Malus domestica 4CL* sequence; https://www.ncbi.nlm.nih.gov/nuccore/XM_008365460), or reported in Supplementary Information file. Source data are provided with this paper.

## Source data

Source Data
