# Supplementary material for: Tailoring poplar lignin without yield penalty by combining a null and haploinsufficient CINNAMOYL-CoA REDUCTASE2 allele
Source: Nat Commun. 2020 Oct 6;11:5020. doi: 10.1038/s41467-020-18822-w (PMC7538556; doi:10.1038/s41467-020-18822-w)
Supplement: Supplementary file 1 — Supplementary Information [file 41467_2020_18822_MOESM1_ESM.pdf]

Tailoring poplar lignin without yield penalty by combining a null and haploinsufficient *CINNAMOYL-CoA REDUCTASE2* allele

De Meester *et al.*

a

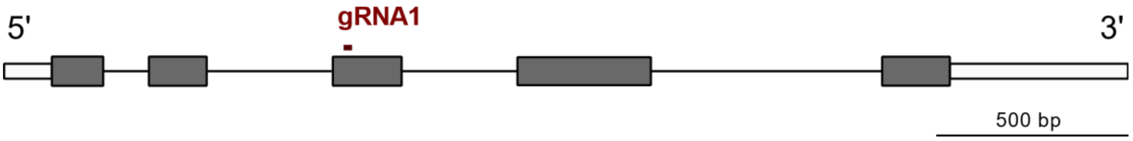

b

Line	Target sequence (N20**NGG**)	Indel
**Dwarfed lines**		
CCR2(-/-) line 1	GCAGTGAACGGGACCAAAAATGTGATCAATTGCGGCGG<br>GCAGTGAACGGGACCAAAAATGTGATCAATTGCGGCGG	+1<br>+1
CCR2(-/-) line 2	GCAGTGAACGGGACCAAAAATGTGATC-.TTGCGGCGG<br>GCAGTGAACGGGACCAAAAATGTGAT--.TTGCGGCGG	-1<br>-2
CCR2(-/-) line 3	GCAGTGAACGGGACCAAAAATGTGATCATTTGCGGCGG<br>GCAGTGAACGGGACCAAAAATGTGATCACTTGCGGCGG	+1<br>+1
CCR2(-/-) line 4	GCAGTGAACGGGACCAAAAATGTGAT--.--GCGGCGG<br>GCAGTGAACGGGACCAAAAATGTGATCAATTGCGGCGG	-4<br>+1
CCR2(-/-) line 5	GCAGTGAACGGGACCAAAAATGTGAT--.TTGCGGCGG<br>GCAGTGAACGGGACCAAAAATGT-----.---CGGCGG	-2<br>-8
CCR2(-/-) line 6	GCAGTGAACGGGACCAAAAATGTGATCATTTGCGGCGG<br>GCAGTGAACGGGACCAAAAATGTGATCA.---------	+1<br>-14
CCR2(-/-) line 7	GCAGTGAACGGGACCAAAAATGTGATC-.TTGCGGCGG<br>GCAGTGAACGGGACCAAAAATGTGAT--.-----GCGG	-1<br>-7
CCR2(-/-) line 8	GCAGTGAACGGGACCAAAAATGTGATCAATTGCGGCGG<br>GCAGTGAACGGGACCAAAAATGTGATCAATTGCGGCGG	+1<br>+1
CCR2(-/-) line 9	GCAGTGAACGGGACCAAAAATGTGATC-.TTGCGGCGG<br>GCAGTGAACGGGACCAAAAATGTGATCAATTGCGGCGG	-1<br>+1
CCR2(-/-) line 10	GCAGTGAACGGGACCAAAAATGTGATCAATTGCGGCGG<br>GCAGTGAACGGGACCAAAAATGTGATCAATTGCGGCGG	+1<br>+1
CCR2(-/-) line 11	GCAGTGAACGGGACCAAAAATGTGATCAATTGCGGCGG<br>GCAGTGAACGGGACCAAAAATGTGATC-.TTGCGGCGG	+1<br>-1
CCR2(-/-) line 13	GCAGTGAACGGGACCAAAAATGTGATCAATTGCGGCGG<br>GCAGTGAACGGGACCAAAAAT-------.---------	+1<br>-29
CCR2(-/-) line 14	GCAGTGAACGGGACCAAAAATGTGATCAATTGCGGCGG<br>GCAGTGAACGGGACCAAAAATGTGATC-.TTGCGGCGG	+1<br>-1
CCR2(-/-) line 15	GCAGTGAACGGGACCAAAAATGTGATCAATTGCGGCGG<br>GCAGTGAACGGGACCAAAAATGTGATC-.TTGCGGCGG	+1<br>-1
CCR2(-/-) line 16	GCAGTGAACGGGACCAAAAATGTGATC-.TTGCGGCGG<br>GCAGTGAACGGGACCAAAAATGTG----.TTGCGGCGG	-1<br>-4
CCR2(-/-) line 17	GCAGTGAACGGGACCAAAAATGTGATCACTTGCGGCGG<br>GCAGTGAACGGGACCAAAAA--------.TTGCGGCGG	+1<br>-8
CCR2(-/-) line 18	GCAGTGAACGGGACCAAAAATGTGA---.-TGCGGCGG<br>GCAGTGAACGGGACCAAAAATGTGATCATTTGCGGCGG	-4<br>+1
CCR2(-/-) line 19	GCAGTGAACGGGACCAAAAATGTGATCAATTGCGGCGG<br>GCAGTGAACGGGACCAAAAATG------.---------	+1<br>-25

CCR2(-/-) line 20	GCAGTGAACGGGACCAAAAATGTG----.---CGGCGG<br>GCAGTGAACGGGACCAAAAATGTGATCATTTGCGGCGG	-7<br>+1
CCR2(-/-) line 21	GCAGTGAACGGGACCAAAAATGTGATCAATTGCGGCGG<br>GCAGTGAACGGGACCAAAAATGTGATCATTTGCGGCGG	+1<br>+1
**Normal-sized line**		
CCR2(-/*) line 12	GCAGTGAACGGGACCAAAAATGTGATCAATTGCGGCGG<br>GCAGTGAACGGGACCAAAAATGTGATCA.---CGGCGG	+1<br>-3
**Wild type**		
P. tremula	GCAGTGAACGGGACCAAAAATGTGATCA.TTG**CGG**CGG	0
P. alba	GCAGTGAACGGGACCAAAAATGTGATCA.TTG**CGG**CGG	0

Supplementary Figure 1. Overview of the targeted *CCR2* locus in *P. tremula* x *P. alba* in which biallelic mutations were desired. (a) Gene model indicating the target of gRNA1 in the *CCR2* alleles of poplar. White boxes, UTRs; grey boxes, exons; black lines, introns. (b) Sequence information of the targeted *CCR2* locus in poplar. For each poplar line, the target sequences of the CRISPR/Cas9 construct are shown. Both alleles (the upper sequence is the *P. tremula CCR2* allele and the lower sequence is the *P. alba CCR2* allele) and the indel patterns are shown. gRNA1 (underlined) and protospacer adjacent motif (PAM; bold text) sequences are highlighted for the wild type. All lines were biallelically edited in the *CCR2* locus. The status of the *CCR2* alleles present in *P. tremula x P. alba* is denoted between the parentheses; the first one represents that of the *P. tremula* allele, the second one that of the *P. alba* allele; -, knock-out; *, protein-modified.

Populus alba **CCR2 wild-type protein sequence**

```
MPVDASSLSGQGQTICVTGAGGFIASWMVKLLLDKGYTVRGTARNPADPKNSHLRELEGAQERLTLCKADLLDYE
SLKEAIQGCDGVFHTASPVTDDPEEMVEPAVNGTKNVIIAAAEAKVRRVVFTSSIGAVYMDPNKGPDVVIDESCW
SDLEFCKNTKNWYCYGKAVAEQAAWDMAKEKGVDLVVVNPVLVLGPLLQPTVNASIVHILKYLTGSAKTYANSVQ
AYVHVRDVALAHILVFETPSASGRYLCSESVLHRGEVVEILAKFFPEYPIPTKCSDEKNPRKQPYKFSNQKLRDL
GFEFTPVKQCLYETVKSLQERGHLPIPKQAAEESLKIQ
```

Populus alba **CCR2 mutated (as in *CCR2*(-/*) line 12) protein sequence**

```
MPVDASSLSGQGQTICVTGAGGFIASWMVKLLLDKGYTVRGTARNPADPKNSHLRELEGAQERLTLCKADLLDYE
SLKEAIQGCDGVFHTASPVTDDPEEMVEPAVNGTKNVI-TAAEAKVRRVVFTSSIGAVYMDPNKGPDVVIDESCW
SDLEFCKNTKNWYCYGKAVAEQAAWDMAKEKGVDLVVVNPVLVLGPLLQPTVNASIVHILKYLTGSAKTYANSVQ
AYVHVRDVALAHILVFETPSASGRYLCSESVLHRGEVVEILAKFFPEYPIPTKCSDEKNPRKQPYKFSNQKLRDL
GFEFTPVKQCLYETVKSLQERGHLPIPKQAAEESLKIQ
```

Supplementary Figure 2. Protein sequence corresponding to the mutated *P. alba CCR2* allele in *CCR2*(-/*) line 12 poplars (*P. tremula x P. alba*). The amino acids that are changed between the wild-type and mutated *P. alba* CCR2 protein are indicated in red.

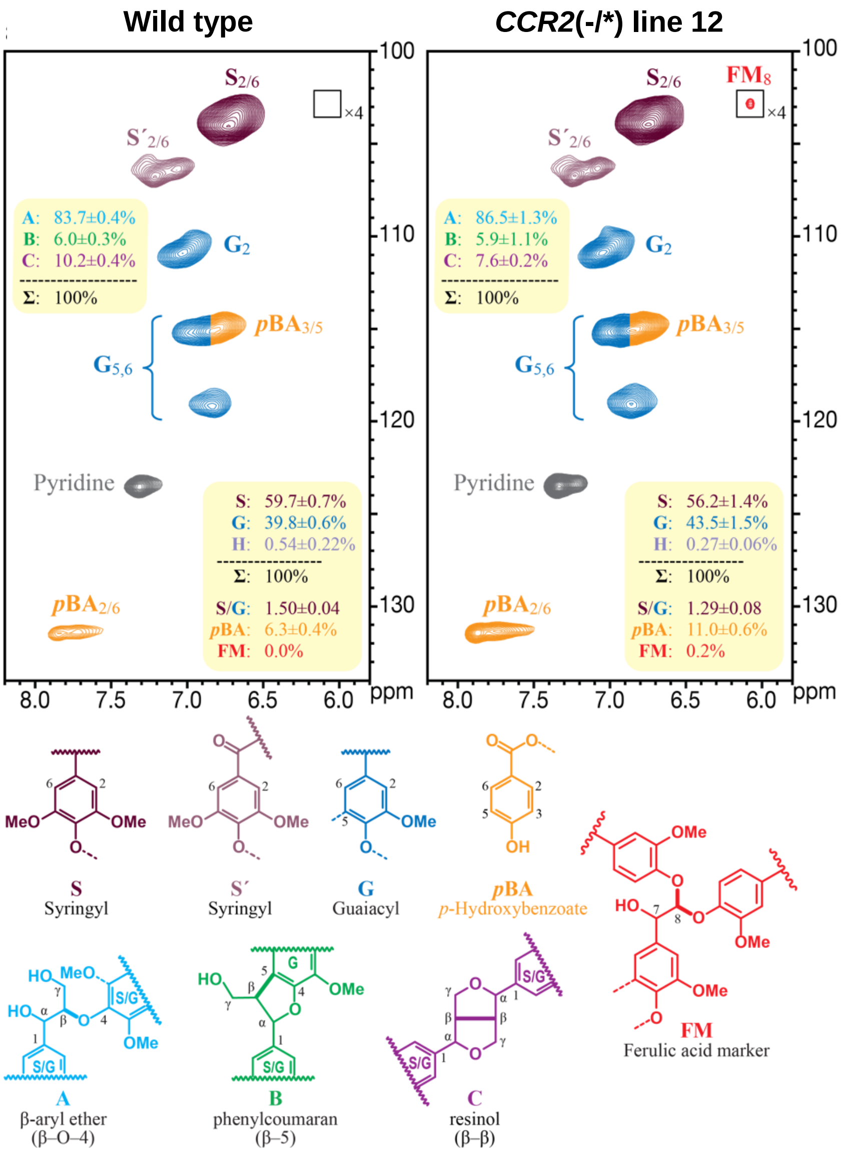

Supplementary Figure 3. Structural characterization of lignin by NMR. Partial short-range ^{1}H–^{13}C correlation (HSQC) NMR spectra (aromatic regions) of extract-free, ball-milled, whole-cell-wall material swelled in DMSO-d_6:pyridine-d_5 (4:1 v/v). Volume-integrals (means ± standard deviation) are given for the lignin aromatic and substructure units that are labeled and color-coded to match their assignments in the spectrum. The aromatic percentages are on a S+G+H=100% basis and the sidechain (not shown) percentages are on an A+B+C=100% basis. *p*BA (*p*-hydroxybenzoate) and FM (ferulate marker) levels are given on an S+G+H=100% basis; note that *p*BA levels are over-represented because they are slowly-relaxing pendent groups on the lignin sidechain. Source data are provided as a Source Data file.

a

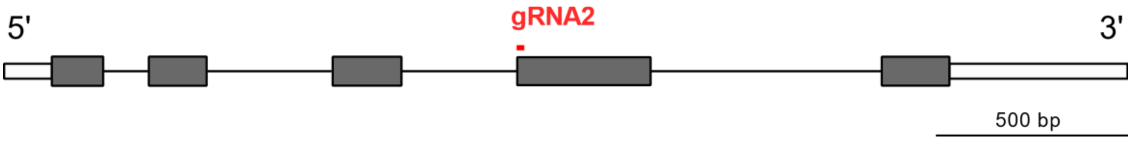

b

Line	Target sequence (N20NGG)	Indel
**Normal-sized lines**		
CCR2(+/-) line 101	CAGAATTGGTATTGCTATGGAA.AGGCTGTGG<br>CAGAATTGGTATTGCTATGG--.---CGGTGG	0<br>-5
CCR2(+/-) line 105	CAGAATTGGTATTGCTATGGAA.AGGCTGTGG<br>CAGAATTGGTATTGC-------.AGGCGGTGG	0<br>-7
CCR2(+/-) line 110	CAGAATTGGTATTGCTATGGAA.AGGCTGTGG<br>CAGAATTGGTATTGCTATGGA-.AGGCGGTGG	0<br>-1
CCR2(+/-) line 113	CAGAATTGGTATTGCTATGGAA.AGGCTGTGG<br>CAGAATTGGTATTGCT----AA.AGGCGGTGG	0<br>-4
CCR2(+/-) line 114	CAGAATTGGTATTGCTATGGAA.AGGCTGTGG<br>CAGAATTGGTATTGCTATGGA-.-------GA	0<br>-8
CCR2(+/-) line 126	CAGAATTGGTATTGCTATGGAA.AGGCTGTGG<br>CAGAATTGGTATTGCTATGGAAAAGGCGGTGG	0<br>+1
**Wild type**		
P. tremula<br>*P. alba*	CAGAATTGGTATTGCTATGGAA.AGGCTGTGG<br>CAGAATTGGTATTGCTATGGAA.AGG**CGG**TGG	0<br>0

Supplementary Figure 4. Overview of the targeted *CCR2* locus in *P. tremula* x *P. alba* in which a monoallelic mutation in the *P. alba* allele was desired. (a) Gene model indicating the target of gRNA2 in the *CCR2* alleles of poplar. White boxes, UTRs; grey boxes, exons; black lines, introns. (b) Sequence information of the targeted *CCR2* locus in poplar. For each poplar line, the target sequences of the CRISPR/Cas9 construct are shown. Both alleles (the upper sequence is the *P. tremula CCR2* allele and the lower sequence is the *P. alba CCR2* allele) and the indel patterns are shown. gRNA2 (underlined) and protospacer adjacent motif (PAM; bold text) sequences are highlighted for the wild type. In *CCR2*(+/-) line 114, also a substitution or insertion (in red) occurred. All lines had a monoallelic mutation in the *CCR2* locus. The status of the *CCR2* alleles present in *P. tremula x P. alba* is denoted between the parentheses; the first one represents that of the *P. tremula* allele, the second one that of the *P. alba* allele; +, wild type; -, knock-out; *, protein-modified.

a

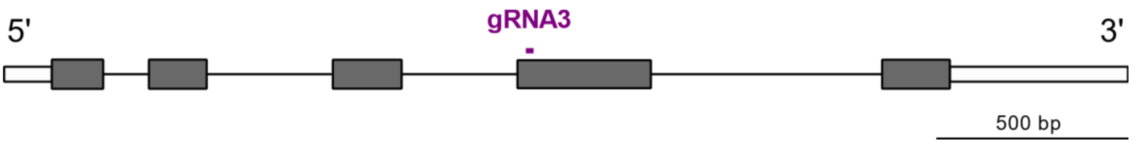

b

Line	Target sequence (N20NGG)	Indel
**Dwarfed line**		
CCR2(-/-) line 202	GGCAGAACAAGCTGCATGGGAATATGGCTAAGGAGAA<br>GGCAGAACAAGCTGC-----.ATATGGCTAAGGAGAA	+1<br>-5
**Intermediate-sized line**		
CCR2(-/*) line 206	GGCAGAACAAGCTGCAT---.--ATGGCTAAGGAGAA<br>GGCAGAACAAGCTGCGT---.ATATGGCTAAGGAGAA	-5<br>-3
**Normal-sized lines**		
CCR2(-/+) line 203	GGCAGAACAAGCTGC----G.ATATGGCTAAGGAGAA<br>GGCAGAACAAGCTGCGTGGG.ATATGGCTAAGGAGAA	-4<br>0
CCR2(-/+) line 204	GGCAGAACAAGCTGCATGGGAATATGGCTAAGGAGAA<br>GGCAGAACAAGCTGCGTGGGA.TATGGCTAAGGAGAA	+1<br>0
CCR2(-/+) line 205	GGCAGAACAAGCTGCAT---.--ATGGCTAAGGAGAA<br>GGCAGAACAAGCTGCGTGGG.ATATGGCTAAGGAGAA	-5<br>0
CCR2(-/+) line 216	GGCAGAACAAGCTGCATG--.ATATGGCTAAGGAGAA<br>GGCAGAACAAGCTGCGTGGG.ATATGGCTAAGGAGAA	-2<br>0
CCR2(-/+) line 219	GGCAGAACAAGCTGCATGGGAATATGGCTAAGGAGAA<br>GGCAGAACAAGCTGCGTGGG.ATATGGCTAAGGAGAA	+1<br>0
CCR2(-/+) line 220	GGCAGAACAAGCTGCATGGGAATATGGCTAAGGAGAA<br>GGCAGAACAAGCTGCGTGGG.ATATGGCTAAGGAGAA	+1<br>0
**Wild type**		
P. tremula<br>*P. alba*	GGCAGAACAAGCTGCATGGG.ATA**TGG**CTAAGGAGAA<br>GGCAGAACAAGCTGCGTGGG.ATATGGCTAAGGAGAA	0<br>0

Supplementary Figure 5. Overview of the targeted *CCR2* locus in *P. tremula* x *P. alba* in which a monoallelic mutation in the *P. tremula* allele was desired. (a) Gene model indicating the target of gRNA3 in the *CCR2* alleles of poplar. White boxes, UTRs; grey boxes, exons; black lines, introns. (b) Sequence information of the targeted *CCR2* locus in poplar. For each poplar line, the target sequences of the CRISPR/Cas9 construct are shown. Both alleles (the upper sequence is the *P. tremula CCR2* allele and the lower sequence is the *P. alba CCR2* allele) and the indel patterns are shown. gRNA3 (underlined) and protospacer adjacent motif (PAM; bold text) sequences are highlighted for the wild type. In *CCR2*(-/-) line 202 and *CCR2*(-/*) line 206, biallelic mutations occurred. All other lines had monoallelic mutations in the *CCR2* locus. The status of the *CCR2* alleles present in *P. tremula x P. alba* is denoted between the parentheses; the first one represents that of the *P. tremula* allele, the second one that of the *P. alba* allele; +, wild type; -, knock-out; *, protein-modified.

1.1 MS/MS sinapic acid *O*-4-hexoside (6.68 min, m/z 385.1136); see De Meester *et al.* (2018)[1]

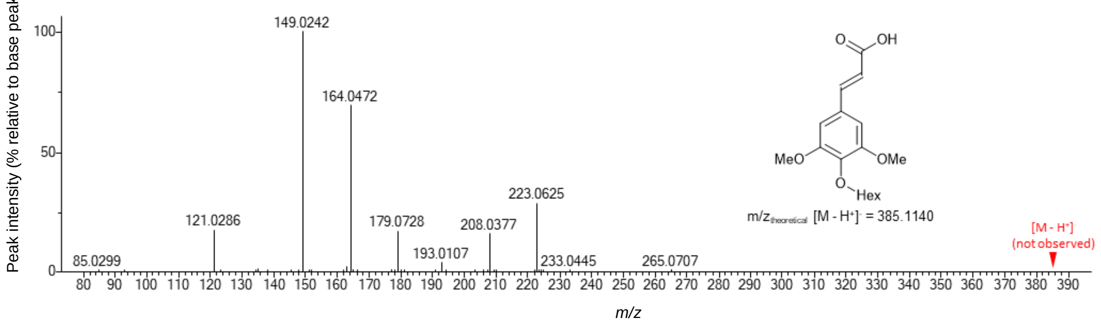

1.2 MS/MS caffeic acid *O*-3/4-hexoside (6.17 min, m/z 341.0876); see De Meester *et al.* (2018)[1]

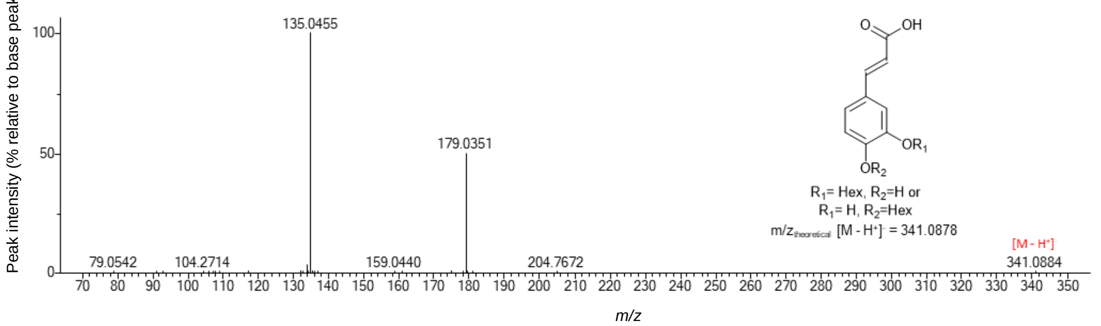

1.3 MS/MS unknown (10.57 min, m/z 653.2085)

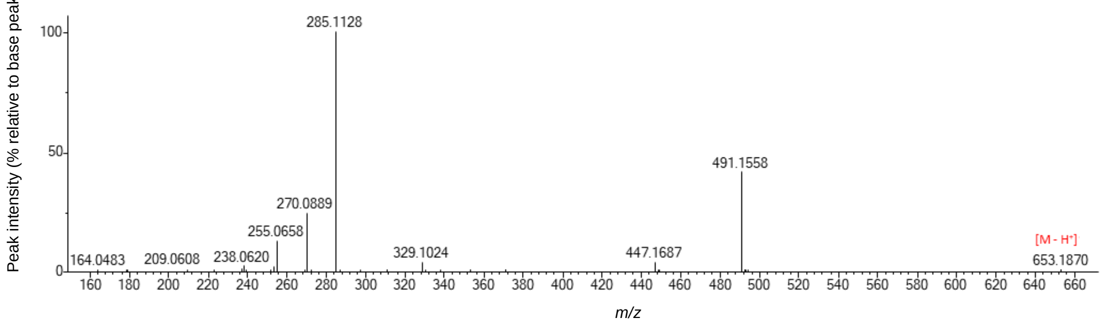

1.4 MS/MS unknown (9.60 min, m/z 815.2251)

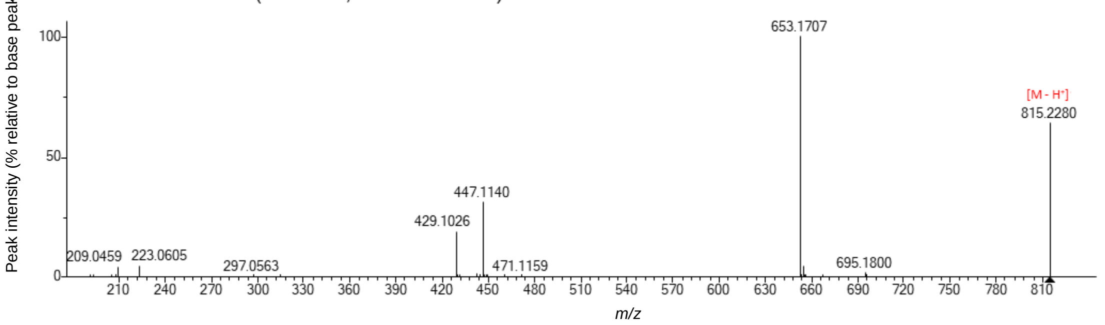

Supplementary Figure 6. MS/MS spectra of the top 10 highest peaks with significantly higher intensities in *CCR2*(-/*) line 12 when compared to the wild type (part 1). The reasoning for the (tentative) structural identification is indicated on the spectra. The number given to each peak corresponds to the one shown in Supplementary Table 4.

1.5 MS/MS ferulic acid + 260 Da 1 (15.05 min, m/z 453.1765)

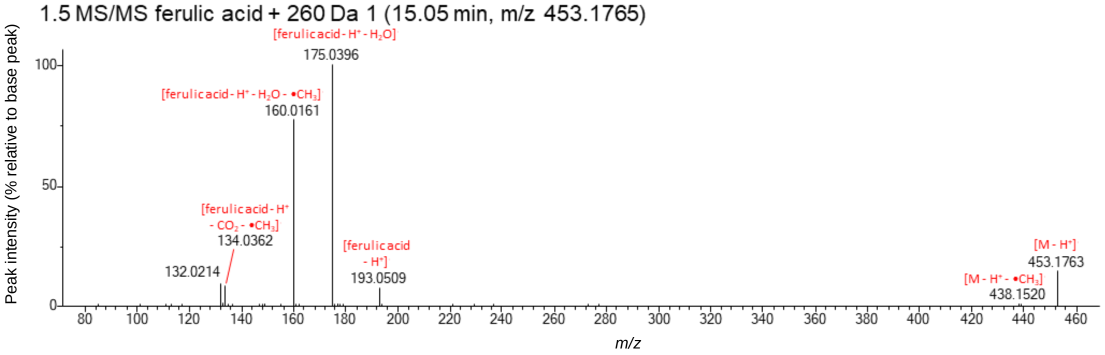

1.6 MS/MS ferulic acid + 260 Da 2 (14.96 min, m/z 453.1765); see compound 1.5

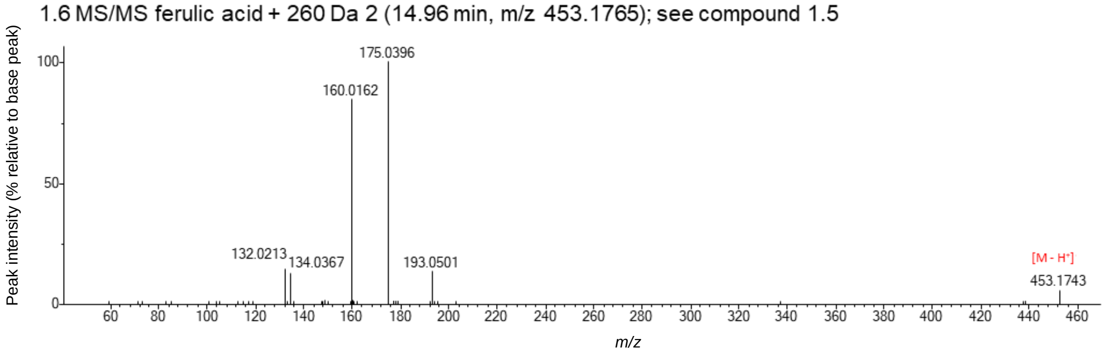

1.7 MS/MS vanillic acid *O*-4-hexoside (3.93 min, m/z 329.0874); see De Meester *et al.* (2018)[1]

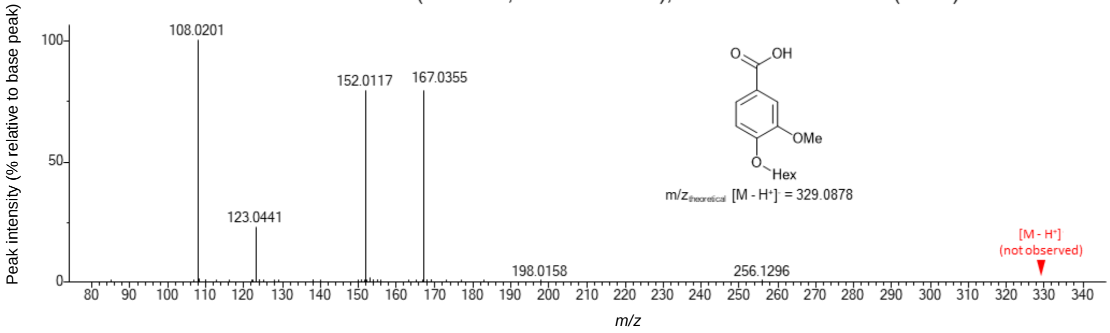

1.8 MS/MS vanillic acid + hexose + sulfate (3.42 min, m/z 409.0443)

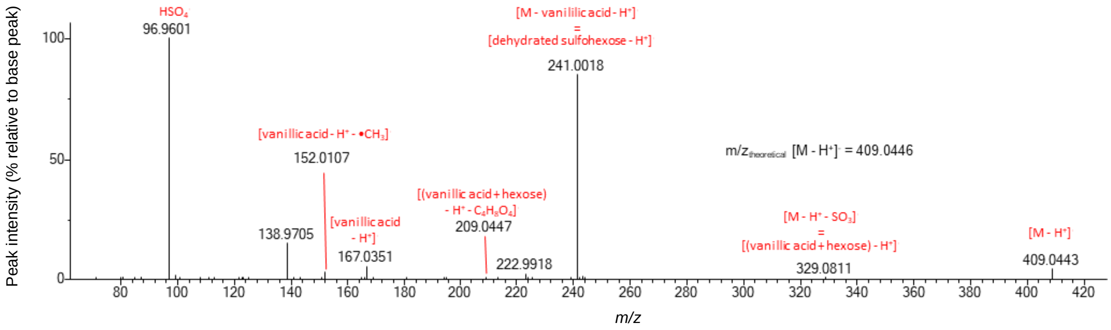

Supplementary Figure 7. MS/MS spectra of the top 10 highest peaks with significantly higher intensities in *CCR2*(-/*) line 12 when compared to the wild type (part 2). The reasoning for the (tentative) structural identification is indicated on the spectra. The number given to each peak corresponds to the one shown in Supplementary Table 4.

1.9 MS/MS unknown (8.49 min, m/z 444.2139)

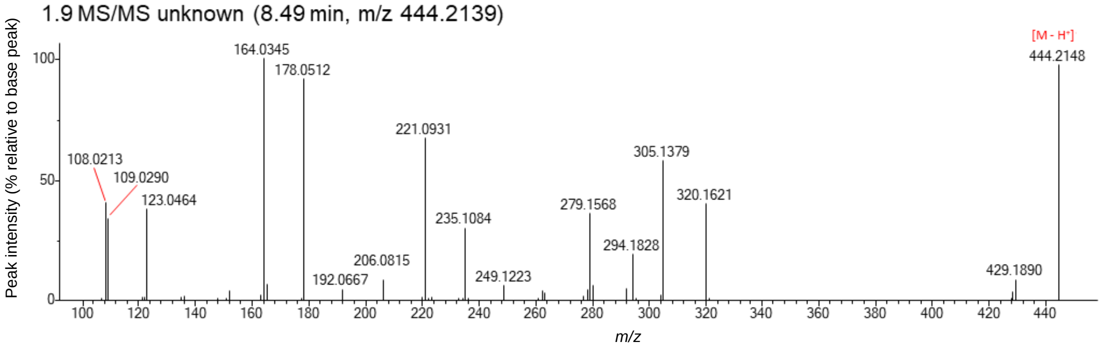

1.10 MS/MS vanillic acid + hexose + 244 Da (16.56 min, m/z 573.1610)

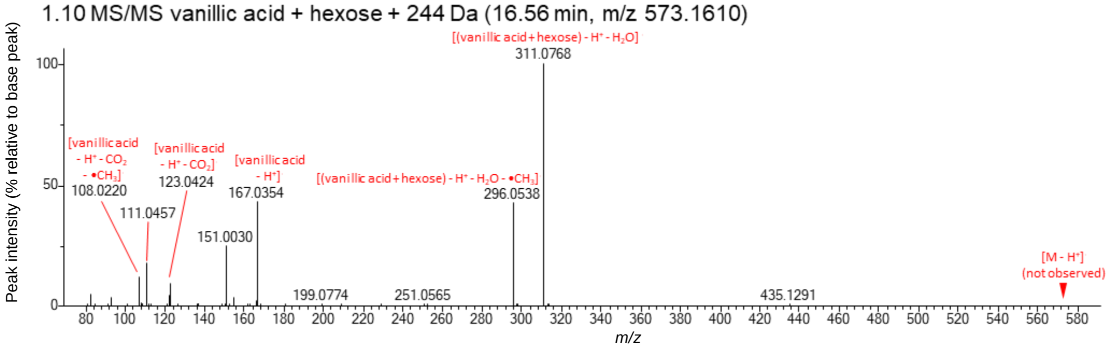

Supplementary Figure 8. MS/MS spectra of the top 10 highest peaks with significantly higher intensities in *CCR2*(-/*) line 12 when compared to the wild type (part 3). The reasoning for the (tentative) structural identification is indicated on the spectra. The number given to each peak corresponds to the one shown in Supplementary Table 4.

2.1 MS/MS G(8-*O*-4)S(8-5)G (16.69 min, m/z 583.2385); see Morreel *et al.* (2010)[2], Vanholme *et al.* (2013)[3]

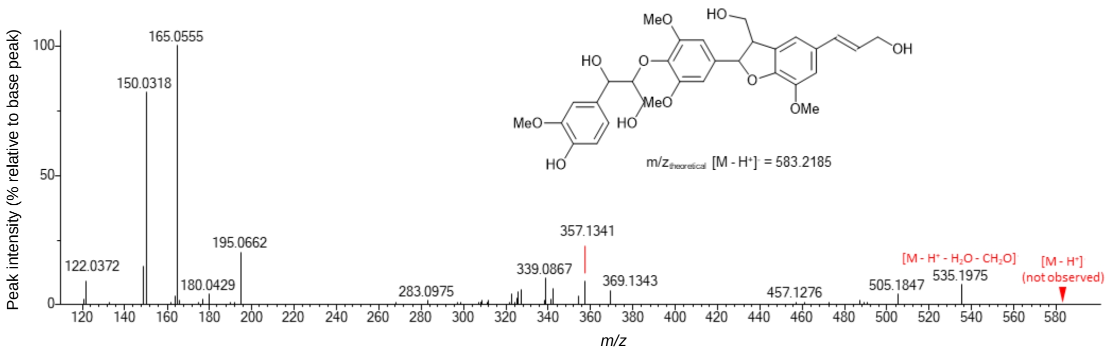

2.2 MS/MS *p*-hydroxybenzoyl S(8-8)S (16.75 min, m/z 555.1868); see Morreel *et al.* (2004)[4]

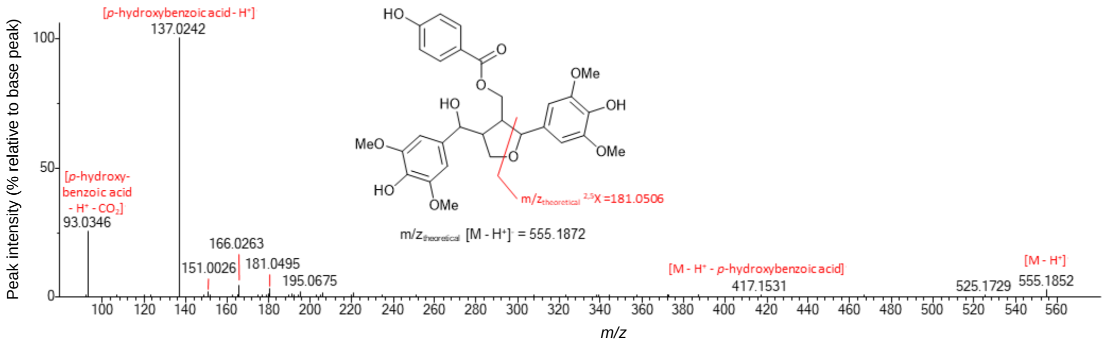

2.3 MS/MS G(8-*O*-4)G(8-*O*-4)S(8-8)S' (17.53 min, m/z 825.2968), see 2.4

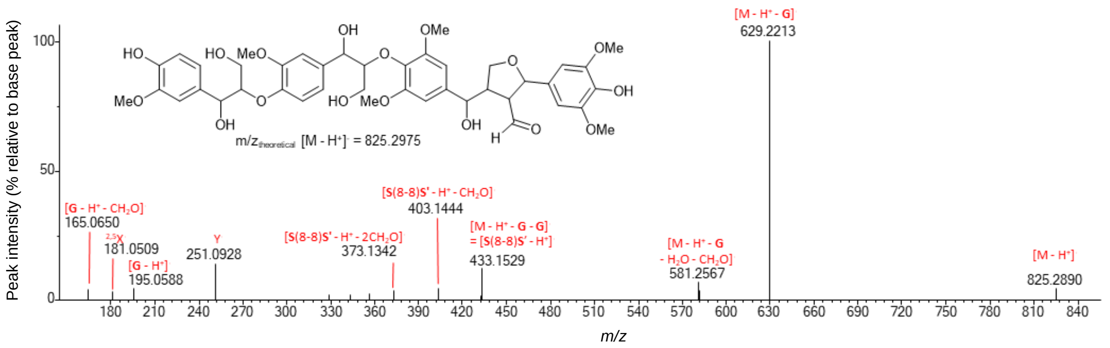

2.4 MS/MS G(8-*O*-4)S(8-8)S' (16.64 min, m/z 629.2233)

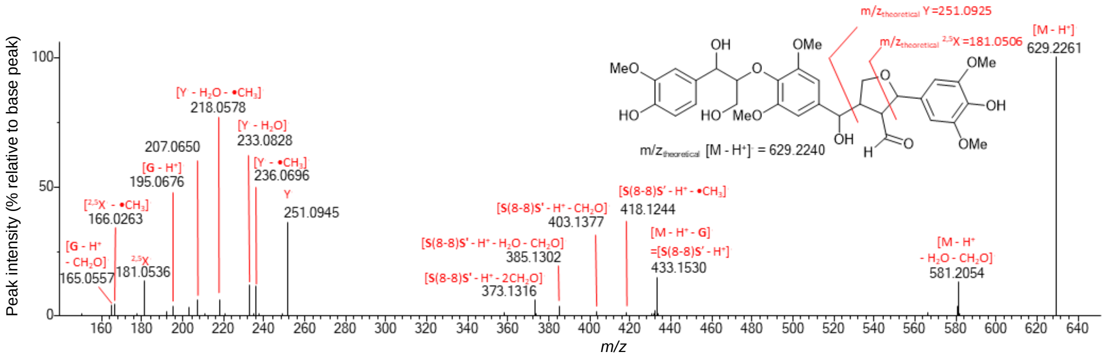

Supplementary Figure 9. MS/MS spectra of the top 10 highest peaks with significantly lower intensities in *CCR2*(-/*) line 12 when compared to the wild type (part 1). The reasoning for the (tentative) structural identification is indicated on the spectra. The number given to each peak corresponds to the one shown in Supplementary Table 4.

2.5 MS/MS unknown (8.64 min, m/z 423.1656)

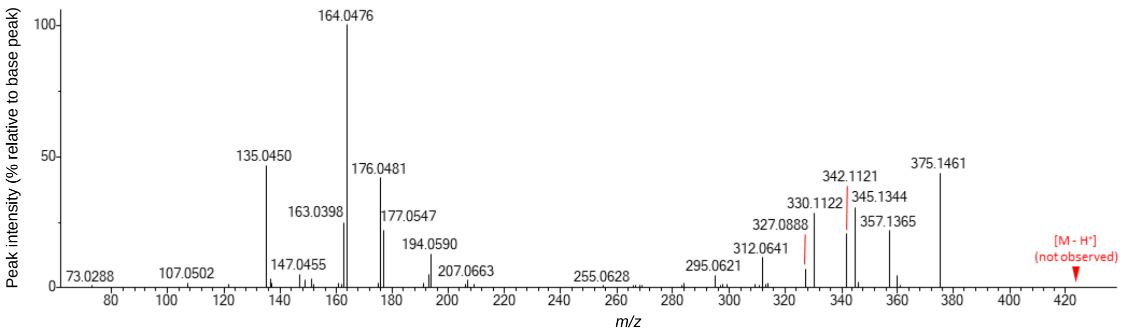

2.6 MS/MS S(8-*O*-4)S(8-5)G (16.48 min, m/z 613.2286); see Morreel *et al.* (2010)[2], Vanholme *et al.* (2013)[3]

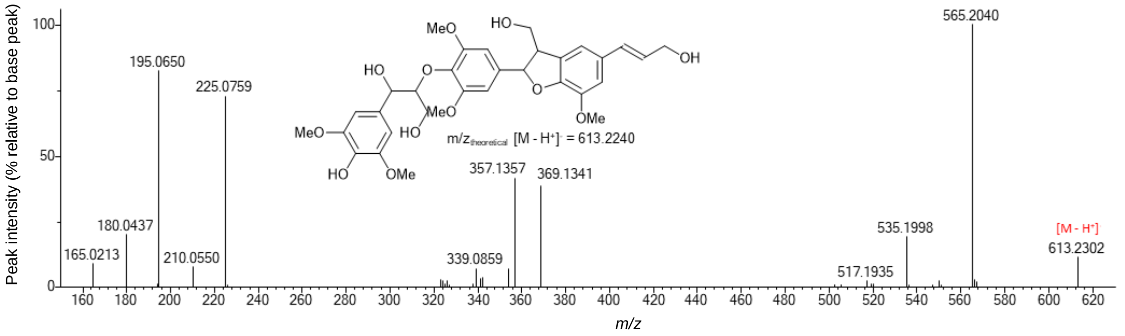

2.7 MS/MS unknown (14.41 min, m/z 433.1499)

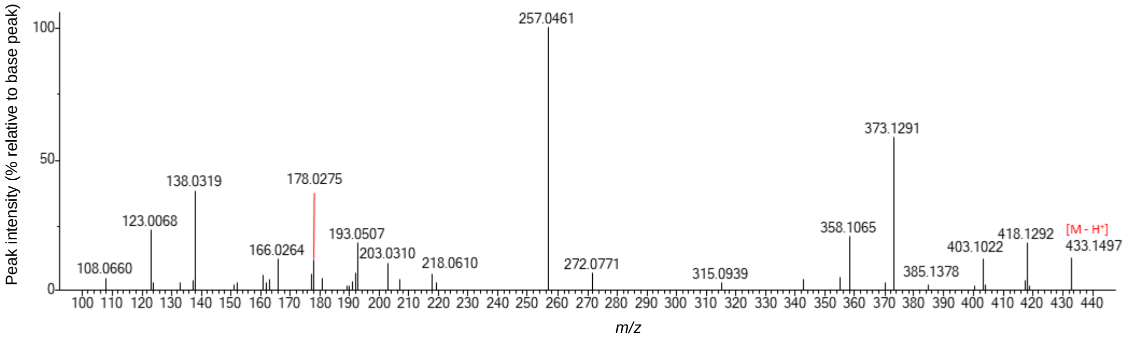

2.8 unknown (16.49 min, m/z 659.2336); no MS/MS available

Supplementary Figure 10. MS/MS spectra of the top 10 highest peaks with significantly lower intensities in *CCR2*(-/*) line 12 when compared to the wild type (part 2). The reasoning for the (tentative) structural identification is indicated on the spectra. The number given to each peak corresponds to the one shown in Supplementary Table 4.

2.9 MS/MS unknown (11.92 min, m/z 683.2186)

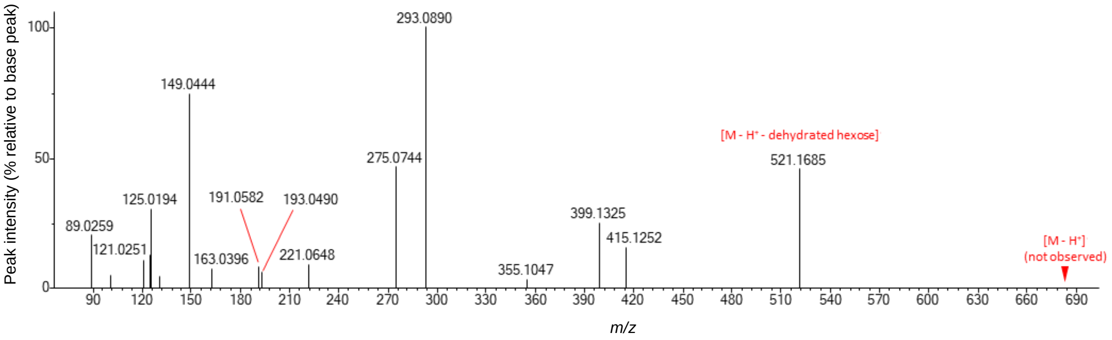

2.10 MS/MS S(8-O-4)S(8-8)S(4-O-8)S (19.26 min, m/z 869.3235)

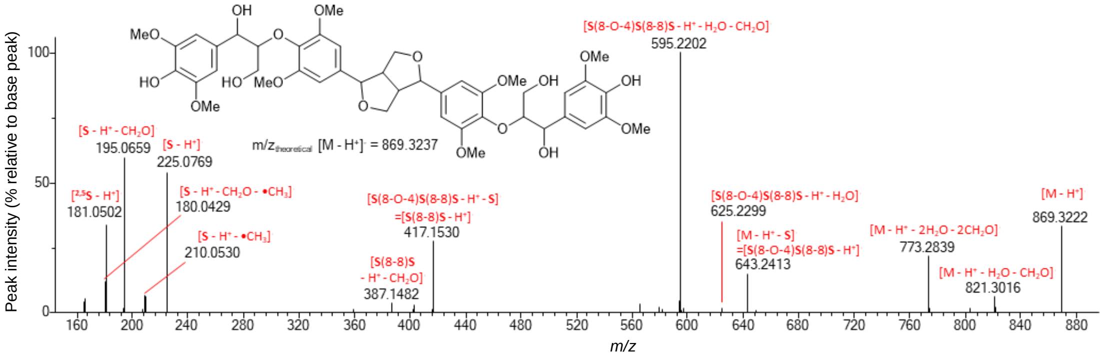

Supplementary Figure 11. MS/MS spectra of the top 10 highest peaks with significantly lower intensities in *CCR2*(-/*) line 12 when compared to the wild type (part 3). The reasoning for the (tentative) structural identification is indicated on the spectra. The number given to each peak corresponds to the one shown in Supplementary Table 4.

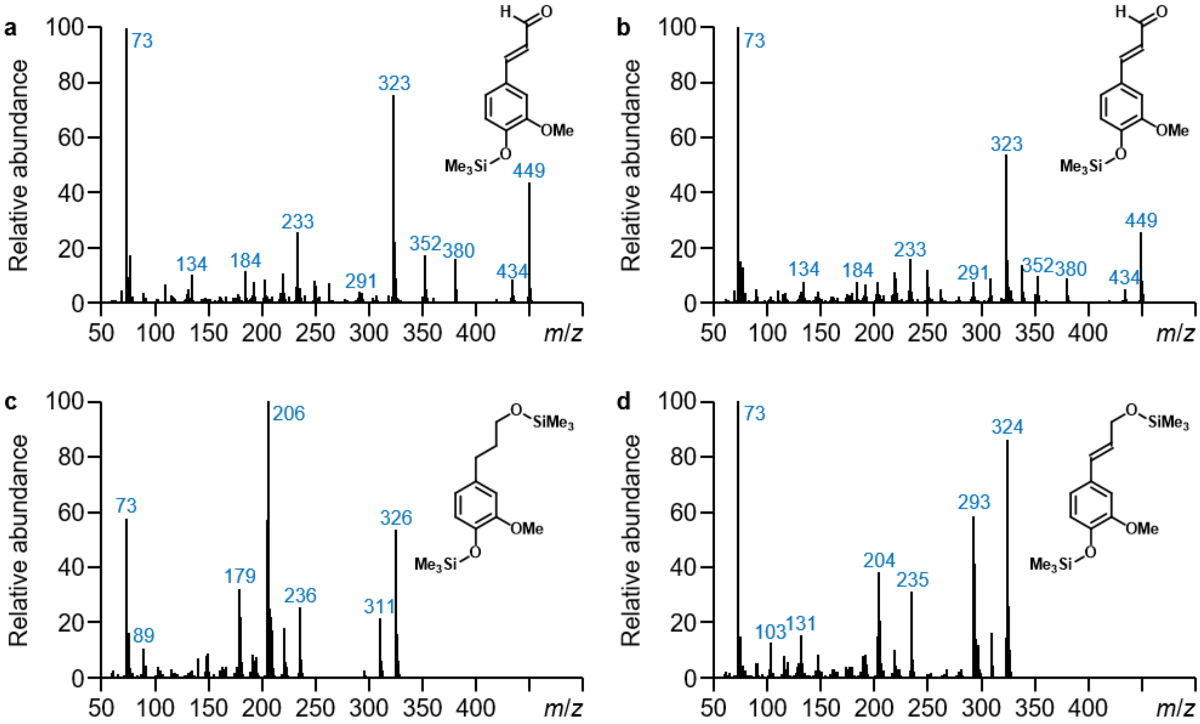

Supplementary Figure 12. EI-MS spectra of trimethylsilylated metabolites produced in the CCR2 yeast activity assay. (a) Authentic coniferaldehyde standard, and products produced in the yeast assay: (b) coniferaldehyde (peak 1 in Figure 8), (c) dihydroconiferyl alcohol (peak 2 in Figure 8), and (d) coniferyl alcohol (peak 3 in Figure 8).

***Populus alba* CCR2 wild-type protein sequence**

```
MPVDASSLSGQGQTICVTGAGGFIASWMVKLLLDKGYTVRGTARNPADPKNSHLRELEGAQERLTLCKADLL
DYESLKEAIQGCDGVFHTASPVTDDPEEMVEPAVNGTKNVIIAAAEAKVRRVVFTSSIGAVYMDPNKGPDVV
IDESCWSDLEFCKNTKNWYCYGKAVAEQAAWDMAKEKGVDLVVVNPVLVLGPLLQPTVNASIVHILKYLTGS
AKTYANSVQAYVHVRDVALAHILVFETPSASGRYLCSESVLHRGEVVEILAKFFPEYPIPTKCSDEKNPRKQ
PYKFSNQKLRDLGFEFTPVKQCLYETVKSLQERGHLPIPKQAAEESLKIQ
```

***Populus alba* CCR2 mutated protein sequence as in *CCR2*(-/*) line 206**

```
MPVDASSLSGQGQTICVTGAGGFIASWMVKLLLDKGYTVRGTARNPADPKNSHLRELEGAQERLTLCKADLL
DYESLKEAIQGCDGVFHTASPVTDDPEEMVEPAVNGTKNVIIAAAEAKVRRVVFTSSIGAVYMDPNKGPDVV
IDESCWSDLEFCKNTKNWYCYGKAVAEQAA-YMAKEKGVDLVVVNPVLVLGPLLQPTVNASIVHILKYLTGS
AKTYANSVQAYVHVRDVALAHILVFETPSASGRYLCSESVLHRGEVVEILAKFFPEYPIPTKCSDEKNPRKQ
PYKFSNQKLRDLGFEFTPVKQCLYETVKSLQERGHLPIPKQAAEESLKIQ
```

Supplementary Figure 13. Protein sequence corresponding to the mutated *P. alba CCR2* allele in *CCR2*(-/*) line 206 poplars (*P. tremula* × *P. alba*). The amino acids that are changed between the wild-type and mutated *CCR2* protein encoded by the *P. alba* allele are indicated in red.

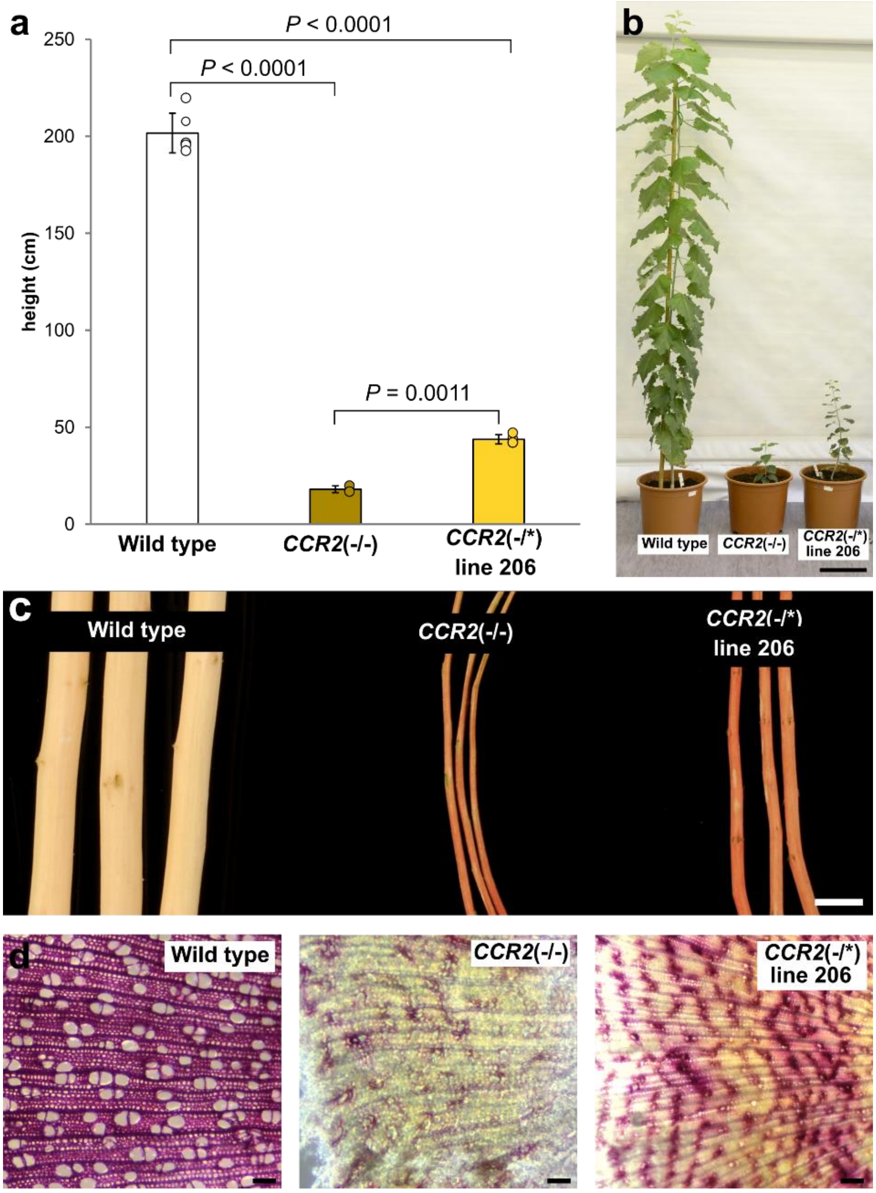

Supplementary Figure 14. Phenotype and xylem morphology of *CCR2*(-/*) line 206. (a) Height of wild type, *CCR2*(-/-), and *CCR2*(-/*) line 206 after growing for 20 weeks in the greenhouse. *CCR2*(-/*) line 206 had a reduced height when compared to wild type, but an increased height when compared to *CCR2*(-/-) (One-way ANOVA with Tukey's post-hoc test, the *P* values are indicated). Individual values (dots) and means (bars) of six biologically independent samples for wild type, three biologically independent samples for *CCR2*(-/-) and five biologically independent samples for *CCR2*(-/*) line 206. Error bars indicate the standard deviation.. (b) Phenotype of representative wild type, *CCR2*(-/-), and *CCR2*(-/*) line 206 grown in the greenhouse for 20 weeks. Scale bar = 20 cm. Wild type, n = 6 biologically independent samples; *CCR2*(-/-), n = 3 biologically independent samples; *CCR2*(-/*) line 206, n = 5 biologically independent samples. (c) Phenotype of representative debarked wild-type, *CCR2*(-/-), and *CCR2*(-/*) line 206 stems grown in the greenhouse for 20 weeks. *CCR2*(-/-) and *CCR2*(-/*) line 206 stems display the red xylem phenotype. Scale bar = 10 mm. Wild type, n = 6 biologically independent samples; *CCR2*(-/-), n = 3 biologically independent samples; *CCR2*(-/*) line 206, n = 5 biologically independent samples. (d) Lignin in cross-sections of stems grown for 20 weeks in the greenhouse visualized using Wiesner staining. Pictures were taken of the xylem tissue, with the pith localized to the right and the epidermis to the left. Images show representative pictures from 5 sections per sample and 3 biologically independent samples per line. Scale bar = 100 µm. The source data underlying Supplementary Figure a4a are provided as a Source Data file.

Yeast codon-optimized *Populus alba* CCR2 wild-type nucleotide sequence

ATGCCGGTTGACGCTAGCAGTTTGTCAGGCCAAGGTCAGACTATCTGCGTTACGGGAGCAGGGGGGTTCATCGCT
TCATGGATGGTAAAGTTATTATTGGATAAGGGGTATACGGTTAGGGGCACGGCCCGTAATCCGGCAGATCCGAAA
AACAGCCATTTGAGGGAATTGGAAGGGGCTCAAGAAAGGCTAACCCTGTGTAAAGCTGACTTGTTAGACTACGAG
AGCTTAAAGGAGGCTATCCAAGGCTGTGATGGTGTGTTCCACACGGCGTCTCCAGTTACTGACGATCCAGAAGAA
ATGGTGGAACCAGCGGTCAATGGAACCAAGAACGTCATAATAGCTGCAGCAGAAGCTAAAGTTAGACGTGTCGTT
TTTACAAGTAGCATCGGCGCTGTATACATGGACCCAAACAAGGGGCCAGATGTCGTGATTGACGAGTCCTGCTGG
TCAGATCTGGAATTTTGCAAAAACACGAAGAATTGGTACTGCTATGGAAAGGCCGTTGCTGAACAGGCAGCATGG
GACATGGCCAAAGAGAAAGGGGTGGATCTTGTCGTCGTAAACCCGGTACTAGTACTGGGCCCCCTGCTTCAGCCC
ACGGTTAATGCGTCCATAGTGCATATCCTAAAGTACTTAACAGGATCTGCCAAAACGTACGCTAATTCTGTTCAA
GCGTATGTACACGTACGTGATGTGGCGCTTGCACACATCCTTGTCTTTGAGACCCCGTCTGCTTCAGGAAGATAC
TTGTGTAGCGAATCAGTGCTGCATAGGGGCGAAGTTGTGGAAATCCTAGCGAAATTCTTTCCCGAGTACCCCATA
CCTACAAAATGTTCAGACGAGAAAAATCCTAGAAAACAGCCTTACAAATTTTCCAACCAGAAATTGAGGGATCTT
GGATTCGAGTTTACGCCTGTAAAACAGTGTTTATACGAAACTGTTAAGAGCCTTCAGGAGAGAGGTCACCTTCCC
ATTCCAAAGCAGGCTGCTGAAGAATCCCTGAAGATTCAATGA

Yeast codon-optimized *Populus alba* CCR2 mutated (as in *CCR2*(-/*) line 12) nucleotide sequence

ATGCCGGTTGACGCTAGCAGTTTGTCAGGCCAAGGTCAGACTATCTGCGTTACGGGAGCAGGGGGGTTCATCGCT
TCATGGATGGTAAAGTTATTATTGGATAAGGGGTATACGGTTAGGGGCACGGCCCGTAATCCGGCAGATCCGAAA
AACAGCCATTTGAGGGAATTGGAAGGGGCTCAAGAAAGGCTAACCCTGTGTAAAGCTGACTTGTTAGACTACGAG
AGCTTAAAGGAGGCTATCCAAGGCTGTGATGGTGTGTTCCACACGGCGTCTCCAGTTACTGACGATCCAGAAGAA
ATGGTGGAACCAGCGGTCAATGGAACCAAGAACGTCATAACTGCAGCAGAAGCTAAAGTTAGACGTGTCGTTTTT
ACAAGTAGCATCGGCGCTGTATACATGGACCCAAACAAGGGGCCAGATGTCGTGATTGACGAGTCCTGCTGGTCA
GATCTGGAATTTTGCAAAAACACGAAGAATTGGTACTGCTATGGAAAGGCCGTTGCTGAACAGGCAGCATGGGAC
ATGGCCAAAGAGAAAGGGGTGGATCTTGTCGTCGTAAACCCGGTACTAGTACTGGGCCCCCTGCTTCAGCCCACG
GTTAATGCGTCCATAGTGCATATCCTAAAGTACTTAACAGGATCTGCCAAAACGTACGCTAATTCTGTTCAAGCG
TATGTACACGTACGTGATGTGGCGCTTGCACACATCCTTGTCTTTGAGACCCCGTCTGCTTCAGGAAGATACTTG
TGTAGCGAATCAGTGCTGCATAGGGGCGAAGTTGTGGAAATCCTAGCGAAATTCTTTCCCGAGTACCCCATACCT
ACAAAATGTTCAGACGAGAAAAATCCTAGAAAACAGCCTTACAAATTTTCCAACCAGAAATTGAGGGATCTTGGA
TTCGAGTTTACGCCTGTAAAACAGTGTTTATACGAAACTGTTAAGAGCCTTCAGGAGAGAGGTCACCTTCCCATT
CCAAAGCAGGCTGCTGAAGAATCCCTGAAGATTCAATGA

Supplementary Figure 15. Sequence information of yeast codon-optimized versions of the wild-type and mutant *P. alba CCR2* alleles used in the CCR2 activity assays in yeast.

Supplementary Table 1. Weekly height of *CCR2*(-/*) line 12.

	**Wild type**	***CCR2*(-/*) line 12**	***P* value**
**Week 8**	58.1 ± 9.1	58.5 ± 6.9	0.920
**Week 9**	65.0 ± 9.6	65.7 ± 7.7	0.849
**Week 10**	73.5 ± 10.4	74.6 ± 8.3	0.785
**Week 11**	82.3 ± 10.8	84.1 ± 8.7	0.678
**Week 12**	91.9 ± 11.3	92.8 ± 8.5	0.835
**Week 13**	103.3 ± 14.2	102.4 ± 8.5	0.855
**Week 14**	112.7 ± 13.6	114.5 ± 9.1	0.729
**Week 15**	127.9 ± 14.1	127.2 ± 9.1	0.890
**Week 16**	144.5 ± 15.4	140.4 ± 10.1	0.472
**Week 17**	158.4 ± 16.4	153.7 ± 10.8	0.445
**Week 18**	175.1 ± 15.4	167.9 ± 11.3	0.233
**Week 19**	192.6 ± 14.4	183.6 ± 11.5	0.129
**Week 20**	207.4 ± 14.2	198.2 ± 11.9	0.123

Height was measured on a weekly basis during a growth period between 8 and 20 weeks in the greenhouse. Values in cm are given as means ± standard deviation. *P* value of the two-tailed Student's *t*-test is given. Wild type, n = 10 biologically independent samples; *CCR2*(-/*) line 12, n = 11 biologically independent samples. Source data are provided as a Source Data file.

Supplementary Table 2. Molecular weight of *CCR2*(-/*) line 12 stem lignin.

<u>Technical repeat 1</u>

Line	Mw (Da)	Mn (Da)	PDI (Mw/Mn)
Wild type	12685	4358	2.91
	14025	4487	3.13
	15453	4860	3.18
CCR2(-/*) line 12	12264	3553	3.45
	11759	3659	3.21

<u>Technical repeat 2</u>

Line	Mw (Da)	Mn (Da)	PDI (Mw/Mn)
Wild type	13823	4477	3.09
	14349	4686	3.06
	14961	4772	3.14
CCR2(-/*) line 12	11934	3459	3.45
	11540	3546	3.25

GPC chromatographic comparison of acetylated lignin from wild-type and *CCR2*(-/*) line 12 stems. Individual values of weight-average molecular weight (Mw), number-average molecular weight (Mn), and polydispersity index (PDI) estimated from the GPC curves (integrated between 80,000 and 600 Da). *CCR2*(-/*) line 12 had a tendency towards a reduced lignin polymerization degree compared to that of the wild type (wild type, n = 3 biologically independent samples; *CCR2*(-/*) line 12, n = 2 biologically independent samples; each biologically independent sample was analyzed twice).

Supplementary Table 3. Saccharification results of *CCR2*(-/*) line 12.

	**Wild type**	***CCR2*(-/*) line 12**	***P* value**
**<u>Glucose yield (%CWR)</u>**			
No pretreatment	9.5 ± 2.1	12.8 ± 1.6	5.9×10^{-4}
Acid pretreatment	12.0 ± 1.6	18.3 ± 1.7	4.0×10^{-8}
Alkaline pretreatment	28.1 ± 3.2	37.9 ± 2.0	8.3×10^{-8}
**<u>Cellulose-to-glucose conversion (%)</u>**			
No pretreatment	23.9 ± 5.4	32.4 ± 4.0	5.1×10^{-4}
Acid pretreatment	30.3 ± 4.1	46.4 ± 4.2	3.4×10^{-8}
Alkaline pretreatment	70.9 ± 8.1	95.9 ± 5.3	6.6×10^{-8}
**<u>Glucose yield per plant (mg)</u>**			
No pretreatment	1571.1 ± 352.8	1969.2 ± 241.4	6.7×10^{-3}
Acid pretreatment	1994.9 ± 268.7	2818.8 ± 255.1	7.6×10^{-7}
Alkaline pretreatment	4663.2 ± 529.9	5824.1 ± 319.1	6.5×10^{-6}

Plants were grown for 20 weeks in the greenhouse. Means ± standard deviation of glucose yield expressed as percentage of cell wall residue (CWR), cellulose-to-glucose conversion efficiency (%), or the glucose yield per plant (based on the dry weight of the stem). Samples were saccharified for 72 h using no pretreatment, acidic pretreatment (1 M HCl), or alkaline pretreatment (62.5 mM NaOH). For all pretreatments tested, *CCR2*(-/*) line 12 had an increased glucose yield compared to the wild type (two-tailed Student's *t*-test, the exact *P* values are shown in the table; wild type, n = 10 biologically independent samples; *CCR2*(-/*) line 12, n = 11 biologically independent samples). Source data are provided as a Source Data file.

Supplementary Table 4. Metabolites with a significantly different abundance in *CCR2*(-/*) line 12 mutants.

no.	retention time (min)	*m/z*	name	Wild type		*CCR2*(-/*) line 12		Fold change *CCR2*(-/*) line 12 vs. wild type	*CCR2*(-/-)		Fold change *CCR2*(-/-) vs. wild type
				mean	SD	mean	SD		mean	SD	
**Compounds with increased abundance in *CCR2*(-/*) line 12 compared to wild type**											
**1.1**	6.69	385.114	sinapic acid *O*-4-hexoside	5944	4187	61926	13048	10.42	**63481**	**16255**	**10.68**
**1.2**	6.17	341.088	caffeic acid *O*-3/4-hexoside	23535	5708	57905	8294	2.46	**116915**	**20521**	**4.97**
**1.3**	10.57	653.208	unknown	508	284	50001	4320	98.40	**39356**	**7776**	**77.45**
**1.4**	9.60	815.225	unknown	523	313	48465	17699	92.64	**12285**	**6705**	**23.48**
**1.5**	15.05	453.177	ferulic acid + 260 Da 1	4156	1057	48073	6790	11.57	**42096**	**12931**	**10.13**
**1.6**	14.96	453.176	ferulic acid + 260 Da 2	13347	8545	41354	6899	3.10	**83131**	**45418**	**6.23**
**1.7**	3.93	329.087	vanillic acid *O*-4-hexoside	8504	2860	37895	10588	4.46	**80384**	**13156**	**9.45**
**1.8**	3.42	409.044	vanillic acid + hexose + sulfate	14553	2638	35978	7205	2.47	22706	5195	1.56
**1.9**	8.49	444.214	unknown	5660	1052	33719	8418	5.96	**141764**	**43628**	**25.05**
**1.10**	16.56	573.161	vanillic acid + hexose + 244 Da	6585	3899	32564	16867	4.94	**59815**	**33656**	**9.08**
**Compounds with decreased abundance in *CCR2*(-/*) line 12 compared to wild type**											
**2.1**	16.69	583.218	G(8-*O*-4)S(8-5)G	69582	25476	31829	5132	0.46	**7078**	**468**	**0.01**
**2.2**	16.75	555.187	*p*-hydroxybenzoyl S(8-8)S	35859	17107	4707	2096	0.13	**120**	**73**	**<0.01**
**2.3**	17.53	825.297	G(8-*O*-4)G(8-*O*-4)S(8-8)S'	20652	9882	6381	2142	0.31	**67**	**79**	**<0.01**
**2.4**	16.64	629.223	G(8-*O*-4)S(8-8)S'	18280	7443	5390	2488	0.29	**515**	**300**	**0.03**
**2.5**	8.64	423.166	unknown	13134	3808	5414	1089	0.41	**1427**	**341**	**0.11**
**2.6**	16.48	613.229	S(8-*O*-4)S(8-5)G	11381	4507	3827	539	0.34	**120**	**88**	**0.01**
**2.7**	14.41	433.150	unknown	9001	3826	2356	888	0.26	**497**	**364**	**0.06**
**2.8**	16.49	659.234	unknown	8766	3571	2081	1100	0.24	**949**	**419**	**0.11**
	8.64	375.144	fragment of **2.5**	8453	3316	2856	465	0.34	**499**	**123**	**0.06**
**2.9**	11.92	683.219	unknown	8414	3709	2929	1273	0.35	**471**	**148**	**0.06**

The top ten most intense peaks in *CCR2*(-/*) line 12 and wild type of which the intensity was either higher or lower, respectively, in *CCR2*(-/*) line 12 compared to the wild type is given. For each compound, average peak areas ± standard deviation (SD) of wild-type (n = 6 biologically independent samples), *CCR2*(-/*) line 12 (n = 6 biologically independent samples), and *CCR2*(-/-) (n = 5 biologically independent samples) samples are given. The mean and SD of *CCR2(-/-)* are given in bold for compounds that also had a significantly different abundance in *CCR2*(-/-) samples when compared to the wild type. For the structural elucidation of the compounds, see Supplementary Figures 6-11. Filters used to select significantly differential peaks: fold change > 2; $P < 0.01$, One-way ANOVA with Dunnett's post-hoc test. Source data are provided as a Source Data file.

Supplementary Note 1. The phenotypes of *CCR2*(-/*) line 12 can be attributed to the specific mutations present in the *CCR2* alleles

While reviewing our manuscript, the referees were concerned that the observed phenotypes in *CCR2*(-/*) line 12 (lignin alterations, red xylem phenotype, normal growth) might be the consequence of off-target mutations. Our reply (and corresponding changes made to the manuscript) convinced the referees of our claims and is shown below.

(A) *In silico* prediction of off-targets. The theoretical possibility of off-targets is extremely low. (i) Off-target mutation in another *CCR* gene family member which might explain the typical CCR-deficient phenotypes such as lower lignin amount, red xylem phenotype and increased incorporation of ferulic acid in the lignin, is excluded by our *in silico* analysis: the eight closest homologs of *CCR2* in *P. tremula x P. alba* (Potri.001G046100, Potri.001G046400, Potri.001G045500, Potri.001G045100, Potri.001G045900, Potri.001G045800, Potri.001G045000 and Potri.T134100) have 3 or 4 mismatches with gRNA1 (5'-GACCAAAAATGTGATCATTG-3') and, more importantly, also lack the subsequent and necessary NGG PAM sequence to allow genome editing in their respective homologous target regions. (ii) The chance of having an off-target mutation in other regions of the *Populus tremula x P. alba* genome is also very unlikely by itself. As off-target effects decrease when the number of mismatches increases, most genome editors consider 4 or more mismatches between the gRNA and any genomic sequence too much to be considered as potential off-targets[5,6]. Following this rule, *in silico* analysis predicted gRNA1 to have only one region in the genome of *P. tremula x P. alba* that could potentially be considered as an off-target (i.e., < 4 mismatches and presence of PAM sequence). However, this region still contains 3 mismatches with gRNA1 and, moreover, because it is intergenic (between Potri.001G282700 and Potri.001G282800), even a possible off-target mutation of this region would be very unlikely to lead to the typical CCR-deficient phenotypes described above. We conclude that, based on our *in silico* analysis, it is highly unlikely that the phenotypes observed in *CCR2*(-/*) line 12 are caused by an off-target mutation.

(B) Experimental validation to exclude off-targets. To support our claims, we have analyzed additional *CCR2* mutant lines. (i) Next to the eight lines described in the first version of the manuscript, we identified 13 additional *CCR2* mutant lines similarly generated using gRNA1. These lines were added to the manuscript. Of these in total 21 *CCR2* mutant lines, all 20 *CCR2*(-/-) bi-allelic knock-out mutants were severely dwarfed. By contrast, *CCR2*(-/*) line 12 grew normally. Similarly, also *CCR2*(-/-) line 202, generated using gRNA3, was severely dwarfed. (ii) Next to *CCR2*(-/*) line 12, we also added the analysis of another *CCR2*(-/*) line generated using a different gRNA (gRNA3) to the manuscript. *CCR2*(-/*) line 206 contained a similar indel pattern in *CCR2* as line 12 (+1/-3), albeit in different positions as those in the *CCR2* alleles in line 12. This *CCR2*(-/*) line 206 provides additional evidence for the claim that a small amino acid modification can alter the activity of CCR2 (to different extents, dependent on the specific modification), thereby also affecting the lignin amount and growth to different extents. More specifically, *CCR2*(-/*) line 206, that was generated using a different gRNA (gRNA3), thus with potentially different off targets, had a biomass yield and lignin amount in between that of wild type and *CCR2*(-/-) lines. Thus, in conclusion, all 21 *CCR2*(-/-) mutants (20 obtained with gRNA1 and 1 with gRNA3) were severely dwarfed, while the 2 *CCR2*(-/*) mutants (one obtained with gRNA1 and one with gRNA3) grew taller than *CCR2*(-/-) knock-outs. The chance that only those 2 better-growing lines with a 3 bp deletion in one allele (that were generated using different gRNAs with potentially different off-targets) also have a (hypothetical) mutation in an off-target gene, while the 21 dwarfed *CCR2*(-/-) (that are generated using the same constructs and gRNAs) do not, is extremely low (P = 0.00395, Fisher exact test with 2x2 contingency table).

(C) The observations are completely in line with literature. Finally, the observed phenotypes are completely in line with the current knowledge about the link between lignin and biomass yield. Lignin levels can be reduced without effect on plant growth, until a certain critical level. If lignin drops below this level, effects on growth (and many other pleiotropic effects) will become apparent. This

phenomenon is described elaborately in literature. We discovered that *CCR2*(-/*) line 12 had a mildly reduced lignin amount (i.e., not below the critical level). Biomass yield was therefore not affected in *CCR2*(-/*) line 12. This is the most likely explanation for the observed phenotype. By contrast, the lignin amount in *CCR2*(-/*) line 206, which is higher than that of *CCR2*(-/-), but still significantly reduced when compared to that of wild type, has dropped below the critical level and is therefore insufficient to maintain a normal growth phenotype. To better frame our finding, we added a paragraph to the discussion describing the link between CCR2 activity (and the amino acid changes), lignin amount and growth, and discuss what is known about this issue in literature.

Supplementary References

1. De Meester, B. *et al.* Vessel-specific reintroduction of CINNAMOYL-COA REDUCTASE1 (CCR1) in dwarfed *ccr1* mutants restores vessel and xylary fiber integrity and increases biomass. *Plant Physiol.* **176**, 611-633 (2018).
2. Morreel, K. *et al.* Mass spectrometry-based fragmentation as an identification tool in lignomics. *Anal. Chem.* **82**, 8095-8105 (2010).
3. Vanholme, B. *et al*. Breeding with rare defective alleles (BRDA): A natural Populus nigra HCT mutant with modified lignin as a case study. *New Phytologist* **198**, 765-776 (2013).
4. Morreel, K. *et al.* Profiling of Oligolignols Reveals Monolignol Coupling Conditions in Lignifying Poplar Xylem. *Plant Physiology* **136**, 3537-3549 (2004).
5. Anderson *et al.* Systematic analysis of CRISPR-Cas9 mismatch tolerance reveals low levels of off-target activity. *J Biotechnol* **211**, 56-65 (2015).
6. Li *et al.* Battling CRISPR-Cas9 off-target genome editing. *Cell Biol Toxicol* **35**, 403-406 (2019).
