# Supplementary material for: Tailoring poplar lignin without yield penalty by combining a null and haploinsufficient CINNAMOYL-CoA REDUCTASE2 allele
Source: Nat Commun. 2020 Oct 6;11:5020. doi: 10.1038/s41467-020-18822-w (PMC7538556; doi:10.1038/s41467-020-18822-w)
Supplement: Supplementary file 4 — Description of Additional Supplementary Files [file 41467_2020_18822_MOESM4_ESM.pdf]

Description of Additional Supplementary Files

File name: Supplementary Dataset 1

Description: Characteristics and intensities of the UHPLC-detected peaks. For each of the 6182 peaks, the m/z, retention time, collision cross section (CCS), peak width, isotope distribution, their intensity in each of the analysed samples, and statistical parameters are given.
