# Supplementary material for: Tailoring poplar lignin without yield penalty by combining a null and haploinsufficient CINNAMOYL-CoA REDUCTASE2 allele
Source: Nat Commun. 2020 Oct 6;11:5020. doi: 10.1038/s41467-020-18822-w (PMC7538556; doi:10.1038/s41467-020-18822-w)
Supplement: Supplementary file 2 — Peer Review [file 41467_2020_18822_MOESM2_ESM.pdf]

Editorial Note: This manuscript has been previously reviewed at another journal that is not operating a transparent peer review scheme. This document only contains reviewer comments and rebuttal letters for versions considered at *Nature Communications.*

REVIEWERS' COMMENTS:

Reviewer #1 (Remarks to the Author):

This is a significant improvement over the previous version. The thorough responses and efforts for performing additional experiments to address reviewers' concerns are much appreciated. With respect to the specific concerns I raised previously, I particularly appreciate the additional results on the generation and characterization of additional CRISPR/Cas9-lines, and the metabolite profiling.

I was also asked to evaluate authors' responses to the reviewer 2 since the reviewer 2 was unavailable to evaluate it this time. Overall, the authors have adequately addressed those specific comments raised by the reviewer 2. The microscopic characterizations are particularly helpful.

That being said, my key concern (also shared by the reviewer 2 on the basis of previous review) remains. In theory, this engineering/screening strategy (the key focus point emphasized by the authors) is doable but in reality, its predictivity and robustness is very low limiting its application. I guess we cannot answer all questions once, so my final evaluation is that this work is scientifically sound with certain degree of novelty and limited application potential.
Jin-Gui Chen

Reviewer #3 (Remarks to the Author):

I thank the authors for addressing the comments of all the reviewers in such detail. I think that the additional data and modifications to the text improve the manuscript substantially, and, in addition to the submission to the new journal, I now think that the paper is suitable for publication.

I note that there are a few minor typos that should be corrected in proofing e.g. MQ-H2O (miliq water?) is used without an explanation.

Reviewer #4 (Remarks to the Author):

The authors have done an exemplary job of responding to reviewer comments to the previous submission of the paper. The additional examination of line 206 addresses the concern about line 12 being caused by an off-target mutation or other coincidence. The modifications to the text and additional data add to, or at least do not detract, from the central story of using "weak" alleles in biotechnology. I would encourage the authors to consider publishing the previous reviews and responses, since some of their analyses and logic, especially with regard to off targets only appears in the review text. Alternatively, the bioinformatics and comments could be added to the supplemental materials.
I noticed one small typo: Line 177: composition instead of compositions

Laura Bartley

REVIEWERS' COMMENTS:

Reviewer #1 (Remarks to the Author):

This is a significant improvement over the previous version. The thorough responses and efforts for performing additional experiments to address reviewers' concerns are much appreciated. With respect to the specific concerns I raised previously, I particularly appreciate the additional results on the generation and characterization of additional CRISPR/Cas9-lines, and the metabolite profiling.

I was also asked to evaluate authors' responses to the reviewer 2 since the reviewer 2 was unavailable to evaluate it this time. Overall, the authors have adequately addressed those specific comments raised by the reviewer 2. The microscopic characterizations are particularly helpful.

That being said, my key concern (also shared by the reviewer 2 on the basis of previous review) remains. In theory, this engineering/screening strategy (the key focus point emphasized by the authors) is doable but in reality, its predictivity and robustness is very low limiting its application. I guess we cannot answer all questions once, so my final evaluation is that this work is scientifically sound with certain degree of novelty and limited application potential.
Jin-Gui Chen

Thank you!

Reviewer #3 (Remarks to the Author):

I thank the authors for addressing the comments of all the reviewers in such detail. I think that the additional data and modifications to the text improve the manuscript substantially, and, in addition to the submission to the new journal, I now think that the paper is suitable for publication.

I note that there are a few minor typos that should be corrected in proofing e.g. MQ-H2O (miliq water?) is used without an explanation.

Thank you, indeed, MQ-H2O is milliQ-water. This is now altered in the text.

Reviewer #4 (Remarks to the Author):

The authors have done an exemplary job of responding to reviewer comments to the previous submission of the paper. The additional examination of line 206 addresses the concern about line 12 being caused by an off-target mutation or other coincidence. The modifications to the text and additional data add to, or at least do not detract, from the central story of using "weak" alleles in biotechnology. I would encourage the authors to consider publishing the previous reviews and responses, since some of their analyses and logic, especially with regard to off targets only appears in the review text. Alternatively, the bioinformatics and comments could be added to the supplemental materials.

Thank you. Indeed, we fully agree that our logic and analysis concerning the off-target issue would be very valuable to add to the paper. Therefore, we have allowed “Transparent peer review” which allows publication of the reviewers comments and author rebuttals.

I noticed one small typo: Line 177: composition instead of compositions

Thank you, this has been altered in the text.

Laura Bartley

We thank the reviewers once more for their valuable contributions to the manuscript.
